# A concise and efficient synthesis of benzimidazo[1,2-*c*]quinazolines through CuI-catalyzed intramolecular *N*-arylations

**DOI:** 10.3762/bjoc.11.258

**Published:** 2015-11-30

**Authors:** Xinlong Pang, Chao Chen, Ming Li, Chanjuan Xi

**Affiliations:** 1 Key Laboratory of Bioorganic Phosphorus Chemistry & Chemical Biology (Ministry of Education), Department of Chemistry, Tsinghua University, Beijing 100084, China, Tel: +86-10-62773684; 2 College of Chemistry and Molecular Engineering, Qingdao University of Science and Technology, Qingdao, 266042, China

**Keywords:** benzimidazo[1,2-*c*]quinazoline, (bromophenyl)iodonium salt, copper catalyst, *o*-cyanoaniline, quinazolin-4(3*H*)-imine, Ullmann N-arylation

## Abstract

A series of functionalized benzimidazo[1,2-*c*]quinazoline derivatives was obtained in excellent yields under mild conditions through a CuI-catalyzed Ullmann *N*-arylation starting from easily available starting materials.

## Introduction

**Scheme 1:**
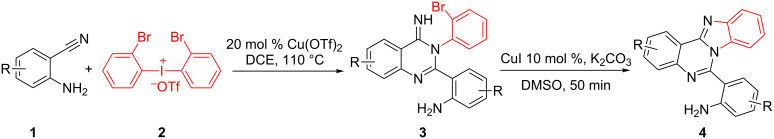
CuI-catalyzed synthesis of benzimidazo[1,2-*c*]quinazolines **4** by intramolecular *N*-arylation of bromo-substituted quinazolin-4(3*H*)-imine derivatives **3**.

Nitrogen-containing heterocycles are ubiquitous backbones in natural products, medicine and organic materials. In addition, they are also important ligands for catalytic reactions. Recently, the conjugation of different types of azaheterocycles in the same molecule has received considerable attention since the resulting ring-fused molecules often show unique organic optoelectronic properties and bioactive activities [1–2]. Among them, benzimidazo[1,2-*c*]quinazolines were intensively investigated and promising biological activities were observed, such as anticancer, antiviral, antimicrobial, anti-inflammatory and anticonvulsant [3–5]. Indeed, some of them are already used as antimicrobial agents and lipid peroxidation inhibitors [6]. Consequently, the development of an efficient way to prepare various benzimidazo[1,2-*c*]quinazoline derivatives is highly desired. Although some methods for the synthesis of benzimidazo[1,2-*c*]quinazoline derivatives have been reported quite recently [7–12], they often require complicated starting materials that are not readily available and need harsh conditions. Herein we report a CuI-catalyzed concise and efficient method for the synthesis of benzimidazo[1,2-*c*]quinazoline derivatives through the intramolecular *N*-arylation reaction of bromo-substituted quinazolin-4(3*H*)-imines that are easily prepared from *o*-cyanoaniline (**1**) and diaryliodonium salts **2** based on our previously published method [13–14] (Scheme 1).

## Results and Discussion

During the study of the synthesis of various carbocycles or heterocycles with copper catalysts [13–17], we found an interesting tandem reaction of *o*-cyanoanilines **1** and diaryliodonium salts **2** to produce quinazolin-4(3*H*)-imine derivatives **3** with Cu(OTf)_2_ as the catalyst [13]. Encouraged by this finding, we initially attempted the reaction of *o*-cyanoaniline (**1a**) with di-(*o*-bromophenyl)iodonium salt **2**. The reaction of 2 equiv of *o*-cyanoaniline (**1a**) with **2** in DCE at 110 °C for 6 h in the presence of 20 mol % Cu(OTf)_2_ bromo-substituted quinazolin-4(3*H*)-imine derivative **3a** in 82% isolated yield. The subsequent treatment of **3a** with CuI (0.1 equiv) and K_2_CO_3_ (1 equiv) in DMSO at room temperature for 50 min led to benzimidazo[1,2-*c*]quinazoline derivative **4a** in 37% yield (Table 1, entry 1). To optimize the yield of the desired product **4a** different conditions were screened. When the reaction temperature was increased to 60 °C, compound **4a** was formed in 98% yield (96% isolated, Table 1, entry 3). On the other hand, the replacement of DMSO by other solvents led to lower yields of **4a** even at elevated temperatures (Table 1, entries 5–9). Other copper salts such as Cu(OTf)_2_, CuBr or CuCl were also able to catalyze the reaction, but they were not as efficient as CuI as the catalyst (Table 1, entries 5–9). It is worth mentioning that the imino group (sp^2^) other than the amino group (sp^3^) in **3a** reacted through the Cu-catalyzed Ullmann reaction [18–25].

**Table 1:** Optimization of reaction conditions for the synthesis of benzimidazo[1,2-c]quinazoline **4a** from quinazolin-4(3*H*)-imine derivative **3a**.

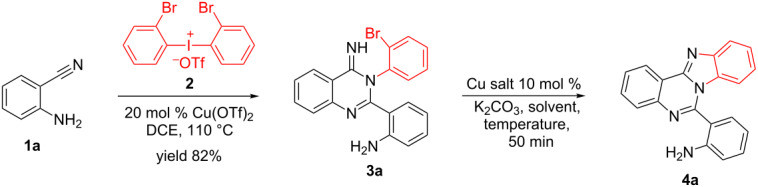				

Entry	Cu salt	Temperature (°C)	Solvent	Yield (%)^a^

1	CuI	rt	DMSO	37
2	CuI	40	DMSO	72
3	CuI	60	DMSO	98 (96^b^)
4	CuI	110	DMSO	98
5	CuI	110	DCE	51
6	CuI	110	CH_3_CN	31
7	CuI	110	DCM	43
8	CuI	110	toluene	89
9	CuI	110	DCE	51
10	Cu(OTf)_2_	60	DMSO	82
11	CuBr	60	DMSO	86
12	CuCl	60	DMSO	91

^a^Estimated crude yield by NMR with trichloroethylene as internal standard. ^b^Isolated yield.

Inspired by the successful cyclization of quinazolin-4(3*H*)-imine **3a**, further imines were prepared and subjected to the cyclization conditions. Notably, in this protocol, after work-up, the desired bromo-substituted quinazolin-4(3*H*)-imine derivatives **3** were directly employed in the next step reaction without the need for chromatographic purification and the results are summarized in Table 2. Quinazolin-4(3*H*)-imines **3** having methyl, fluoro or chloro substituents all worked well in the reaction and provided the corresponding quinazolines **4** in high yields (Table 2, entries 2, 3 and 6). In addition changing the position of the fluoro substituent did not affect the yield of the products (Table 2, entries 3–5).

**Table 2:** CuI-catalyzed synthesis of benzimidazo[1,2-*c*]quinazolines **4** from bromo-substituted quinazolin-4(3*H*)-imines **3**.

Entry	Bromo-substituted quinazolin-4(3*H*)-imine **3**	Benzimidazo[1,2-*c*]quinazoline **4**	Yield^a^

1	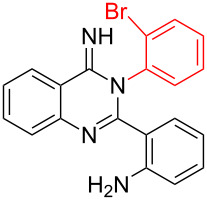 **3a**	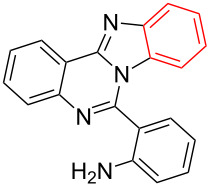 **4a**	96%
2	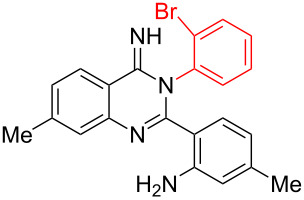 **3b**	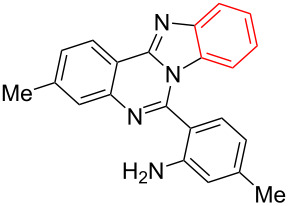 **4b**	95%
3	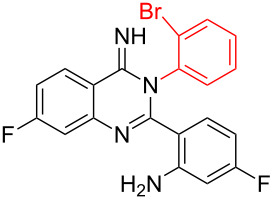 **3c**	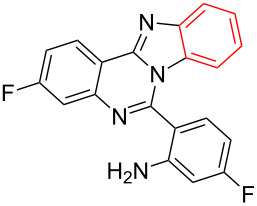 **4c**	95%
4	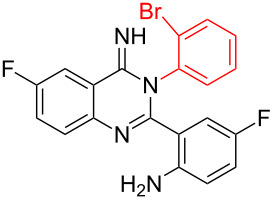 **3d**	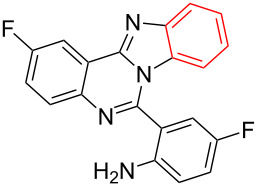 **4d**	94%
5	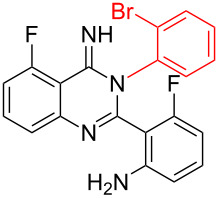 **3e**	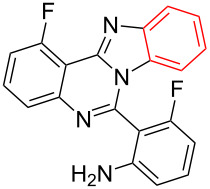 **4e**	93%
6	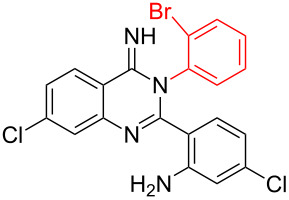 **3f**	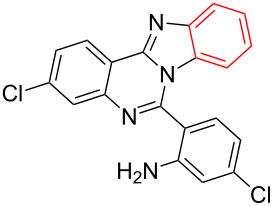 **4f**	96%

^a^Isolated yield.

**Scheme 2:**
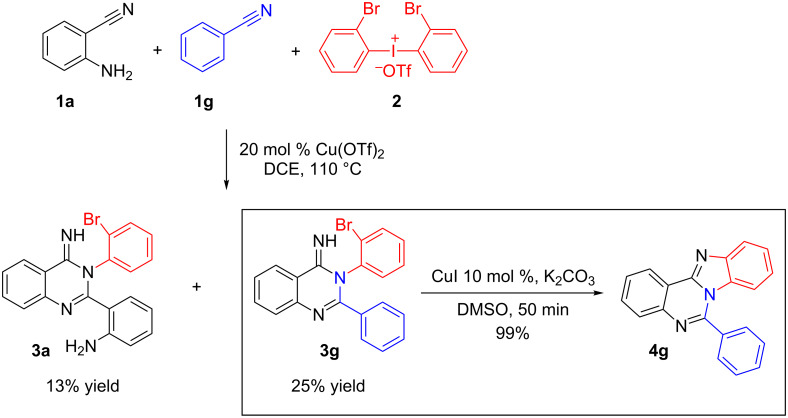
Cu-catalyzed reaction of *o*-cyanoaniline (**1a**), benzonitrile (**1g**) and di-(*o*-bromophenyl)iodonium salt **2** producing imine **3g** and its subsequent cyclization in the presence of CuI.

To further expand the scope of the protocol, we attempted the synthesis of imine **3g** starting from two different nitriles. The reaction of *o*-cyanoaniline (**1a**), benzonitrile (**1g**) and di-(*o*-bromophenyl)iodonium salt **2** in the presence of Cu(OTf)_2_ gave the desired imine **3g** together with imine **3a**. After isolation of **3g** it was further treated with 10 mol % of CuI in DMSO for 50 min to give product **4g** in quantitative yield (Scheme 2).

**Scheme 3:**
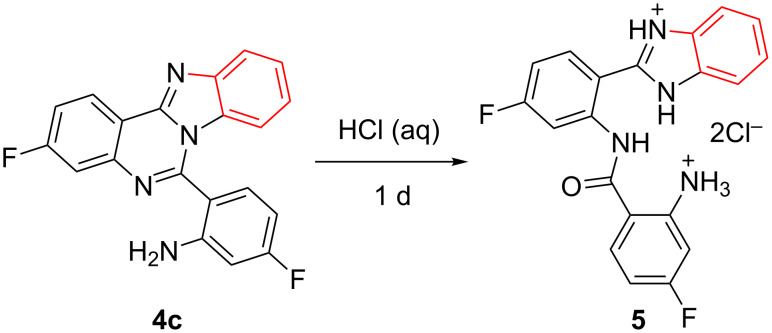
Acid-promoted ring-opening reaction from quinazoline **4c** to **5**.

**Figure 1:**
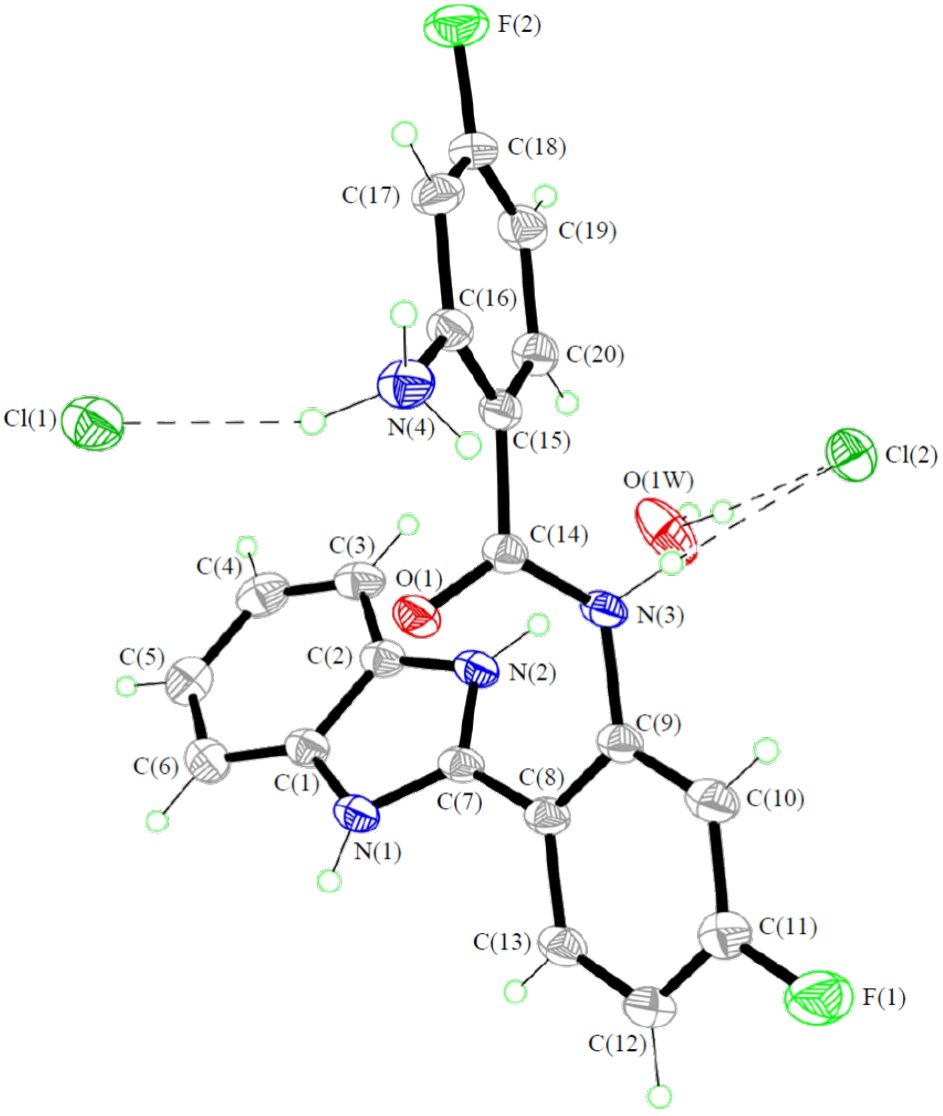
ORTEP drawing of **5**, [C_20_H_16_F_2_N_4_O]·2Cl·H_2_O with 35% probability ellipsoids, showing the atomic numbering scheme.

It is worth mentioning that during the course of our study, we observed that products **4** were not stable to acid. For example, treatment of **4c** with aqueous HCl solution led to ring-opening product **5** (Scheme 3). The structure of **5** was confirmed by X-ray diffraction analysis (Figure 1), clearly showing the cleavage of the quinazoline ring rather than the imidazole ring [26].

## Conclusion

We have demonstrated a CuI-catalyzed pathway to produce functionalized benzimidazo[1,2-*c*]quinazoline derivatives from bromo-substituted quinazolin-4(3*H*)-imines through a selective intramolecular *N*-arylation reaction. The bromo-substituted quinazolin-4(3*H*)-imines are easily synthesized from readily available *o*-cyanoanilines and di-(*o*-bromophenyl)iodonium salt. The extension of the reaction and the investigation of the biological activity of the new products are currently under progress in our laboratory.

## Supporting Information

File 1 Full experimental procedures, characterization data, and NMR charts for compounds **3a–g** and **4a–g**.
