# Supplementary material for: A concise and efficient synthesis of benzimidazo[1,2-c]quinazolines through CuI-catalyzed intramolecular N-arylations
Source: Beilstein J Org Chem. 2015 Nov 30;11:2365–9. doi: 10.3762/bjoc.11.258 (PMC4685915; doi:10.3762/bjoc.11.258)
Supplement: File 1 — Full experimental procedures, characterization data, and NMR charts for compounds 3a–g and 4a–g. [file Beilstein_J_Org_Chem-11-2365-s001.pdf]

Supporting Information

for

A concise and efficient synthesis of benzimidazo[1,2-*c*]quinazolines through CuI-catalyzed intramolecular *N*-arylations

Xinlong Pang[1,2], Chao Chen[1*], Ming Li[2] and Chanjuan Xi[1*]

Address: [1]Key Laboratory of Bioorganic Phosphorus Chemistry & Chemical Biology (Ministry of Education), Department of Chemistry, Tsinghua University, Beijing 100084, China, Tel: +86-10-62773684 and [2]College of Chemistry and Molecular Engineering, Qingdao University of Science and Technology, Qingdao, 266042, China

Email: Chao Chen - chenchao01@mails.tsinghua.edu.cn; Chanjuan Xi - cjxi@tsinghua.edu.cn

*Corresponding author

Full experimental procedures, characterization data, and copies of NMR spectra for compounds **3a–g** and **4a–g**.

Contents:

1). General comments S2
2). Typical procedure for the preparation of **3**... S3
3). Typical procedure for the preparation of **4**... S6
4). NMR spectra of compounds **3a**–**3f** S9
5). NMR spectra of compounds **4a**–**4f** S16
6). References.. S23

1. General Comments

All the reactions were performed in predried screw-capped tubes with a Teflon-lined septum under N_2 atmosphere. Diaryliodonium salts were prepared according to the literature [1-6]. *o*-Cyanoanilines except for those that are commercially available were prepared according to literature procedures [7-10]. All solvents were freshly distilled before use. Column chromatography was performed on silica gel (particle size 10–40 μm, Ocean Chemical Factory of Qingdao, China). ^{1}H NMR and ^{13}C NMR spectra were recorded on a JEOL AL-300 MHz, AL-400 MHz or AL-600 MHz spectrometer at ambient temperature with $CDCl_3$ as the solvent. Chemical shifts (δ) were given in ppm, referenced to the residual proton resonance of the solvents ($CDCl_3$: 7.26 ppm; DMSO-d_6:2.49 ppm), ^{13}C NMR to the carbon resonance of $CDCl_3$ (77.16 ppm) or DMSO-d_6 (39.7 ppm). Coupling constants (*J*) were given in Hertz (Hz). The terms m, dq, q, t, d, s refer to multiplet, doublet quartet, quartet, triplet, doublet, and singlet.

2. Typical procedure for the preparation of 2-(3-(2-bromophenyl)-4-imino-3,4-dihydro-quinazolin-2-yl)aniline (3a).

In a 250 mL sealed tube the mixture of 2-aminobenzonitrile (1.77 g, 15 mmol), diaryliodonium salt (4.39, 7.5 mmol) and CH_2Cl_2 (45 mL) was heated to 110 °C and stirred for 6 h. This was followed by reaction with aqueous K_2CO_3 and standard work-up. Then the reaction mixture was diluted with 150 mL CH_2Cl_2, washed with saturated sodium bicarbonate, extracted with water and CH_2Cl_2, washed with brine, dried over anhydrous magnesium sulfate, and concentrated under reduced pressure to afford the crude product. Purification by flash column chromatography (hexane:ethyl acetate:trimethylamine 300:100:4) afforded 3.64 g (82%) of **3a** as a yellow solid. ^1H NMR (300 MHz, $CDCl_3$) δ 8.24 (s, 1H), 7.72–7.53 (m, 3H), 7.48–7.41 (m, 1H), 7.40–7.32 (m, 1H), 7.26 (dd, *J* = 8.0, 7.2 Hz, 1H), 7.17 (dd, *J* = 7.4, 6.5 Hz, 1H), 7.05–6.91 (m, 2H), 6.59 (d, *J* = 8.1 Hz, 1H), 6.44 (t, *J* = 7.5 Hz, 1H), 4.28 (s, 2H); ^{13}C NMR (75 MHz, $CDCl_3$) δ 153.0, 144.8, 144.0, 133.8, 133.2, 131.0, 130.7, 130.4, 128.9, 128.4, 127.5, 127.3, 125.5(2×C), 124.1, 120.9, 120.6(2×C), 117.6, 116.5; mp 137–138 °C; HRMS–ESI (*m*/*z*): $[M + H]^+$ calcd for $C_{20}H_{15}BrN_4$ 391.2639; found 391.2645; MS–ESI (*m*/*z*): $[M + H]^+$ calcd for $C_{20}H_{15}BrN_4$ 391.3; found, 391.3.

2-(3-(2-Bromophenyl)-4-imino-7-methyl-3,4-dihydroquinazolin-2-yl)-5-methylaniline (3b): ^1H NMR (400 MHz, $CDCl_3$) δ 8.37 (s, 1H), 7.59 (d, *J* = 5.2 Hz, 2H), 7.38 (dd, *J* = 8.2, 5.7, 2.7 Hz, 1H), 7.07 (d, *J* = 7.9 Hz, 1H), 6.98 (s, 1H), 6.90 (dd, *J* = 14.9, 7.6 Hz, 3H), 6.76 (d, *J* = 7.7 Hz, 1H), 6.55 (d, *J* = 8.1 Hz, 1H), 6.36 (t, *J* = 7.5 Hz, 1H), 4.44 (s, 2H), 2.22 (s, 3H), 2.13 (s, 3H); ^{13}C NMR (100 MHz, $CDCl_3$) δ 157.23, 154.51, 145.75, 145.41, 144.83, 141.08, 138.53, 130.89, 130.50, 130.13, 130.06, 129.60, 128.21, 126.61, 119.62, 119.60, 119.39, 118.00, 22.83, 22.45; mp: 139-140 °C; ESI-HRMS: m/z calcd for $C_{20}H_{15}BrN_4$

[M+H]$^+$: 419.0793; found: 418.0791; ESI-MS: m/z calcd for $C_{22}H_{19}BrN_4$[M+H]$^+$: 418.1.; found: 418.1.

2-(3-(2-Bromophenyl)-7-fluoro-4-imino-3,4-dihydroquinazolin-2-yl)-5-fluoroaniline (3c): ^{1}H NMR (400 MHz, $CDCl_3$) δ 8.20 (d, *J* = 8.5 Hz, 1H), 7.52 (d, *J* = 1.7 Hz, 1H), 7.42 – 7.27 (m, 4H), 7.20 – 7.10 (m, 2H), 6.64 (d, *J* = 8.3 Hz, 1H), 6.53 (d, *J* = 1.3 Hz, 1H), 6.34 (dd, *J* = 8.3, 1.6 Hz, 1H), 4.48 (s, 2H); ^{13}C NMR (100 MHz, DMSO-d_6) δ 165.42 (d, *J* = 198.5 Hz), 163.0 (d, *J* = 188.8 Hz), 154.79, 148.1 (d, *J* = 12.2 Hz), 147.3 (d, *J* = 12.5 Hz), 131.8 (d, *J* = 10.7 Hz), 129.52, 129.6 (d, *J* = 10.0 Hz), 114.8 (d, *J* = 22.6 Hz), 112.6 (d, *J* = 21.3 Hz), 101.6 (d, *J* = 22.3 Hz), 100.6 (d, *J* = 24.5 Hz); mp: 144-145 ^{o}C; ESI-HRMS: m/z calcd for $C_{20}H_{13}BrF_2N_4$ [M+H]$^+$: 427.0292; found: 427.0294; ESI-MS: m/z calcd for $C_{20}H_{13}BrF_2N_4$[M+H]$^+$: 427.1; found: 427.1.

2-(3-(2-Bromophenyl)-6-fluoro-4-imino-3,4-dihydroquinazolin-2-yl)-4-fluoroaniline (3d): ^{1}H NMR (400 MHz, DMSO-d_6) δ 7.98 (d, *J* = 8.4 Hz, 1H), 7.57 – 7.47 (m, 2H), 7.38 (d, *J* = 7.4 Hz, 2H), 7.31 (s, 1H), 7.26 (d, *J* = 6.9 Hz, 1H), 6.82 (dd, *J* = 9.3, 2.9 Hz, 1H), 6.66 (dd, *J* = 8.7, 3.0 Hz, 1H), 6.39 (dd, *J* = 8.9, 4.9 Hz, 1H), 5.12 (s, 2H); ^{13}C NMR (100 MHz, DMSO-d_6) δ 160.8 (d, *J* = 243.3 Hz), 154.3 (d, *J* = 231.0 Hz) , 152.14, 142.56, 141.95, 130.0 (d, *J* = 8.2 Hz), 129.67, 129.53, 129.33, 123.4 (d, *J* = 9.8 Hz), 121.02, 120.95, 120.78, 116.7 (d, *J* = 22.1 Hz), 116.22, 116.15, 115.9 (d, *J* = 23.4 Hz), 111.0 (d, *J*= 24.3 Hz); mp: 144-145 ^{o}C; ESI-HRMS: m/z calcd for $C_{20}H_{13}BrF_2N_4$ [M+H]$^+$: 427.0292; found: 427.0294; ESI-MS: m/z calcd for $C_{20}H_{13}BrF_2N_4$[M+H]$^+$: 427.1; found: 427.1.

2-(3-(2-Bromophenyl)-5-fluoro-4-imino-3,4-dihydroquinazolin-2-yl)-3-fluoroaniline (3e): ^{1}H NMR (400 MHz, **$CDCl_3$**) δ 8.00 (s, 1H), 7.57 (dd, *J* = 8.8, 5.1 Hz, 1H), 7.39 – 7.32 (m, 3H), 7.23 (dd, *J* = 7.7, 0.9 Hz, 2H), 6.67 (dd, *J* = 8.5, 2.9 Hz, 1H), 6.51 (dd, *J* = 8.8, 3.7 Hz, 2H), 4.08 (s, 2H); ^{13}C NMR (100 MHz, DMSO-d_6) δ 160.6 (d, *J* = 162.4 Hz), 159.8 (d, *J* = 162.4 Hz), 159.90, 159.3 (d, *J* = 8.9 Hz), 158.42, 151.28, 151.13, 150.28, 148.1 (d, *J* = 8.1 Hz), 147.9 (d, *J* = 8.0 Hz) 147.56, 137.37, 135.76, 135.66, 133.75, 133.64, 131.47, 131.37, 131.09, 130.98, 129.34, 129.13, 129.00, 128.7 (d, *J* = 18.1 Hz), 127.9 (d, *J* = 57.1 Hz), 124.08, 114.31, 114.10, 113.94, 113.72, 111.61, 111.56, 110.71, 110.51, 109.27, 109.07, 108.50, 108.30, 100.8 (d, *J* = 22.4 Hz), 100.6 (d, *J* = 21.4 Hz); mp: 144-145 ^{o}C; ESI-HRMS: m/z calcd for $C_{20}H_{13}BrF_2N_4$ [M+H]$^+$: 427.0292; found: 427.0294; ESI-MS: m/z calcd for

$C_{20}H_{13}BrF_2N_4[M+H]^+$: 427.1; found: 427.1.

2-(3-(2-Bromophenyl)-7-chloro-4-imino-3,4-dihydroquinazolin-2-yl)-5-chloroaniline (3f): ^{1}H NMR (400 MHz, DMSO-d_6) δ 8.32 (s, 1H), 7.78 (d, *J* = 7.3 Hz, 1H), 7.40 (d, *J* = 8.3 Hz, 4H), 7.10 (s, 1H), 6.90 (d, *J* = 8.6 Hz, 1H), 6.33 (d, *J* = 8.6 Hz, 1H), 6.24 (s, 1H), 5.44 (s, 2H); ^{13}C NMR (100 MHz, DMSO-d_6) δ 153.43, 145.21, 144.36(2×C), 135.69, 132.28, 131.90(2×CH), 130.17(2×C), 129.93(2×C), 129.67, 129.49(2×CH), 128.08(2×CH), 122.30, 119.27, 117.05, 105.17; mp: 164-165 ^{o}C; ESI-HRMS: m/z calcd for $C_{20}H_{13}BrCl_2N_4$ $[M+H]^+$: 458.9701; found: 458.9705; ESI-MS: m/z calcd for $C_{20}H_{13}BrCl_2N_4[M+H]^+$: 459.0; found: 459.0.

3-(2-Bromophenyl)-2-phenylquinazolin-4(3H)-imine (3g): ^{1}H NMR (301 MHz, $CDCl_3$) δ 8.26 (d, *J* = 8.0 Hz, 1H), 7.71 – 7.64 (m, 2H), 7.61 – 7.55 (m, 1H), 7.46 (dd, *J* = 5.6, 2.0 Hz, 1H), 7.42 – 7.37 (m, 2H), 7.29 (dd, *J* = 9.7, 7.3 Hz, 3H), 7.19 (dd, *J* = 2.2, 1.4 Hz, 3H); ^{13}C NMR (76 MHz, $CDCl_3$) δ 155.33, 154.26, 144.41, 137.19, 135.41, 134.20, 133.35, 132.26, 130.80, 129.43, 128.81, 128.41(2×C), 127.95(3×C), 127.32, 125.44, 124.20, 120.41, 77.56, 77.14, 76.72; mp: 143-144 ^{o}C; ESI-HRMS: m/z calcd for $C_{20}H_{14}BrN_3$ [M+H]+: 376.0371; found: 376.0371; ESI-MS: m/z calcd for $C_{20}H_{14}BrN_3$[M+H]+: 376.0; found: 376.0.

2. Typical procedure for the preparation of 2-(benzo[4,5]imidazo[1,2-*c*]quinazolin-6-yl)aniline (4a).

In a similar manner as described in reference [14] a mixture of CuI (0.1 mmol, 19.0 mg), K_2CO_3 (1 mmol, 138 mg), 2-(3-(2-bromophenyl)-4-imino-3,4-dihydroquinazolin-2-yl)aniline (**3a**, 1 mmol) were placed in a sealed tube and evacuated and recharged with N_2 for 3 times. After DMSO (1.5 mL) was added, the tube was sealed and the mixture was allowed to stir at 60 °C for 50 min. Afterwards, H_2O (100 mL) was added and the mixture was extracted with EtOAc (5 mL × 3) and the combined the organic extracts were dried over anhydrous Na_2SO_4. Evaporation of the solvent followed by purification on silica gel (petroleum ether:EtOAc:triethylamine 20:20:1 to 300:100:4) afforded 297.6 mg (96%) of **4a** as light yellow solid. ^1H NMR (400 MHz, DMSO-d_6) δ 8.60 (d, *J* = 7.4 Hz, 1H), 7.92 (d, *J* = 7.9 Hz, 1H), 7.84 (dd, *J* = 19.3, 7.9 Hz, 2H), 7.72 (t, *J* = 7.2 Hz, 1H), 7.41 (t, *J* = 7.4 Hz, 1H), 7.34 (t, *J* = 7.5 Hz, 1H), 7.27 (d, *J* = 7.3 Hz, 1H), 7.13 (t, *J* = 7.6 Hz, 1H), 6.85 (d, *J* = 8.1 Hz, 1H), 6.72 (t, *J* = 7.2 Hz, 1H), 6.65 (d, *J* = 8.3 Hz, 1H), 5.31 (s, 2H); ^{13}C NMR (100 MHz, DMSO-d_6) δ 148.24, 148.05, 147.17, 144.38, 143.11, 132.09, 131.94, 129.95, 129.57, 128.44, 128.42, 125.62, 124.18, 122.93, 119.72, 119.16, 118.44, 116.21, 115.78, 114.48; mp 270–271 °C; HRMS–ESI (*m*/*z*): $[M + H]^+$ calcd for $C_{20}H_{14}N_4$ 311.1218; found, 311.1221.

5-Methyl-2-(3-methylbenzo[4,5]imidazo[1,2-*c*]quinazolin-6-yl)aniline (4b): yellow solid, 321.1 mg, yield: 95%. ^1H NMR (400 MHz, DMSO-d_6) δ 8.45 (d, *J* = 8.1 Hz, 1H), 7.83 (d, *J* = 8.0 Hz, 1H), 7.70 (s, 1H), 7.53 (dd, *J* = 8.1, 1.1 Hz, 1H), 7.39 (t, *J* = 7.6 Hz, 1H), 7.16 – 7.09 (m, 2H), 6.72 (d, *J* = 8.3 Hz, 1H), 6.66 (s, 1H), 6.54 (d, *J* = 7.7 Hz, 1H), 5.22 (s, 2H), 2.51 (s, 3H), 2.31 (s, 3H); ^{13}C NMR (100 MHz, DMSO-d_6) δ 148.38, 148.19, 147.09, 144.44, 143.23, 142.26, 141.30, 129.88, 129.72, 129.59, 128.08, 125.50, 124.00, 122.67, 119.52, 117.29,

116.58, 116.09 (2×CH), 114.55, 21.91; mp: 281-282 °C; ESI-HRMS: m/z calcd for $C_{22}H_{18}N_4[M+H]^+$: 339.1513; found: 339.1519.

5-Fluoro-2-(3-fluorobenzo[4,5]imidazo[1,2-*c*]quinazolin-6-yl)aniline (4c): yellow solid, 328.7 mg, yield: 95%. ^1H NMR (400 MHz, DMSO-d_6) δ 7.97 (d, *J* = 8.1 Hz, 1H), 7.88 – 7.78 (m, 2H), 7.62 (dd, *J* = 13.8, 4.3 Hz, 1H), 7.49 (t, *J* = 7.4 Hz, 1H), 7.36 (dd, *J* = 15.3, 8.2 Hz, 1H), 7.25 (t, *J* = 7.7 Hz, 1H), 6.74 (d, *J* = 8.4 Hz, 1H), 6.68 (d, *J* = 8.3 Hz, 1H), 6.54 (t, *J* = 8.8 Hz, 1H), 5.73 (s, 2H); ^{13}C NMR (100 MHz, DMSO-d_6) δ 162.26 (d, *J* = 229 Hz) , 158.76 (d, *J* = 246 Hz), 157.54, 149.42, 149.37, 144.69, 144.55, 143.63, 133.2 (d, *J* = 10.6 Hz), 132.54 (d, *J* = 8.9 Hz), 128.30, 126.13, 124.59, 124.56, 124.10, 120.48, 115.2 (d, J = 19.6 Hz), 113.25, 111.75, 108.9 (d, *J* = 11.2 Hz), 106.0 (d, *J* = 19.8 Hz), 101.9 (d, *J* = 20.7 Hz); mp: 230-231 °C; ESI-HRMS: m/z calcd for $C_{20}H_{12}F_2N_4[M+H]^+$: 347.1030; found: 347.1031.

4-Fluoro-2-(2-fluorobenzo[4,5]imidazo[1,2-*c*]quinazolin-6-yl)aniline (4d): light yellow solid, 325.5 mg, yield: 94%. ^1H NMR (400 MHz, DMSO-d_6) δ 8.63 (dd, *J* = 8.3, 6.4 Hz, 1H), 7.86 (d, *J* = 8.0 Hz, 1H), 7.73 (dd, *J* = 10.0, 2.4 Hz, 1H), 7.59 (dd, *J* = 8.7, 2.3 Hz, 1H), 7.42 (t, *J* = 7.6 Hz, 1H), 7.35 (dd, *J* = 8.2, 6.9 Hz, 1H), 7.19 (t, *J* = 7.8 Hz, 1H), 6.73 (d, *J* = 8.4 Hz, 1H), 6.60 (dd, *J* = 11.8, 2.3 Hz, 1H), 6.52 (dd, *J* = 8.5, 2.3 Hz, 1H), 5.69 (s, 2H); ^{13}C NMR (101 MHz, DMSO-d_6) δ 164.95(d, *J* = 243.7 Hz), 164.32(d, *J* = 248.3 Hz), 149.48(d, *J* = 12.3 Hz), 148.57, 147.87, 144.83(d, *J* = 12.4 Hz), 144.29, 132.18(d, *J* = 11.0 Hz), 129.39, 126.69(d, *J* = 9.9 Hz), 125.82, 123.16, 119.74, 117(d, *J* = 23.7 Hz), 116.16, 114.84, 114.32, 113.69(d, *J* = 21.8 Hz), 103.02(d, *J* = 22.4 Hz), 101.39(d, *J* = 24.6 Hz); mp: 235-236 °C; ESI-HRMS: m/z calcd for $C_{20}H_{12}F_2N_4[M^+H]^+$: 347.1030; found: 347.1031.

3-Fluoro-2-(1-fluorobenzo[4,5]imidazo[1,2-*c*]quinazolin-6-yl)aniline (4e): yellow solid, 321.8 mg, yield: 93%. ^1H NMR (400 MHz, DMSO-d_6) δ 8.31 – 8.23 (m, 1H), 8.00 (dd, *J* = 8.9, 5.1 Hz, 1H), 7.89 (d, *J* = 8.0 Hz, 1H), 7.75 – 7.66 (m, 1H), 7.44 (t, *J* = 7.6 Hz, 1H), 7.22 (dd, *J* = 7.8, 4.3 Hz, 3H), 6.85 (dd, *J* = 9.7, 4.7 Hz, 1H), 6.69 (d, *J* = 8.3 Hz, 1H), 5.23 (s, 2H); ^{13}C NMR (100 MHz, DMSO-d_6) δ 161.36(d, *J* = 246.1 Hz), 154.08(d, *J* = 231.9 Hz), 147.53, 146.11, 144.22, 144.03, 139.87, 131.31(d, *J* = 8.7 Hz), 129.38, 125.88, 123.47, 120.64, 120.54, 120.38(d, *J* = 24.3 Hz), 119.96, 119.04(d, *J* = 22.0 Hz), 118.29(d, *J* = 7.4 Hz), 117(d, *J* = 7.1 Hz), 116.14(d, *J* = 23.8 Hz), 114.33, 109.08(d, *J* = 24.2 Hz); mp: 239-240 °C; ESI-HRMS: m/z calcd for $C_{20}H_{12}F_2N_4[M+H]^+$: 347.1030; found: 347.1031.

5-Chloro-2-(3-chlorobenzo[4,5]imidazo[1,2-*c*]quinazolin-6-yl)aniline (4f): yellow solid, 362.9 mg, yield: 96%. ^{1}H NMR (400 MHz, DMSO-d_6) δ 8.58 (d, *J* = 8.5 Hz, 1H), 8.00 (d, *J* = 1.8 Hz, 1H), 7.89 (d, *J* = 8.1 Hz, 1H), 7.77 (dd, *J* = 8.5, 1.9 Hz, 1H), 7.45 (t, *J* = 7.7 Hz, 1H), 7.33 (d, *J* = 8.1 Hz, 1H), 7.23 (t, *J* = 7.8 Hz, 1H), 6.88 (d, *J* = 1.7 Hz, 1H), 6.74 (dd, *J* = 13.3, 5.1 Hz, 2H), 5.67 (s, 2H); ^{13}C NMR (101 MHz, DMSO-d_6) δ 148.72, 148.40, 147.68, 144.36, 144.03, 136.43, 136.39, 131.80, 129.34, 128.76, 127.63, 125.96, 123.44, 119.93, 118.10, 116.92, 115.75, 114.71, 114.28; mp: 301-302 ^{o}C; ESI-HRMS: m/z calcd for $C_{20}H_{12}Cl_2N_4[M+H]^+$: 379.0439; found: 379.0442.

6-Phenylbenzo[4,5]imidazo[1,2-*c*]quinazoline (4g): white solid, 292.2 mg, yield: 99%. ^{1}H NMR (76 MHz, $CDCl_3$) δ 8.81 – 8.71 (m, 1H), 7.99 (dd, *J* = 7.9, 4.9 Hz, 2H), 7.83 – 7.73 (m, 3H), 7.72 – 7.59 (m, 4H), 7.51 – 7.43 (m, 1H), 7.17 – 7.02 (m, 1H), 6.61 (d, *J* = 8.5 Hz, 1H); ^{13}C NMR (76 MHz, $CDCl_3$) δ 148.59, 148.13, 144.49, 142.48, 134.40, 131.94, 131.06, 129.39(2×CH), 128.45, 128.37, 128.32, 125.69, 124.28, 122.65, 120.07, 118.52, 114.43; mp: 270-271 ^{o}C; ESI-HRMS: m/z calcd for $C_{20}H_{13}N_3[M+H]^+$: 296.1109; found: 296.1109.

4). NMR spectra for compounds 3a–3g

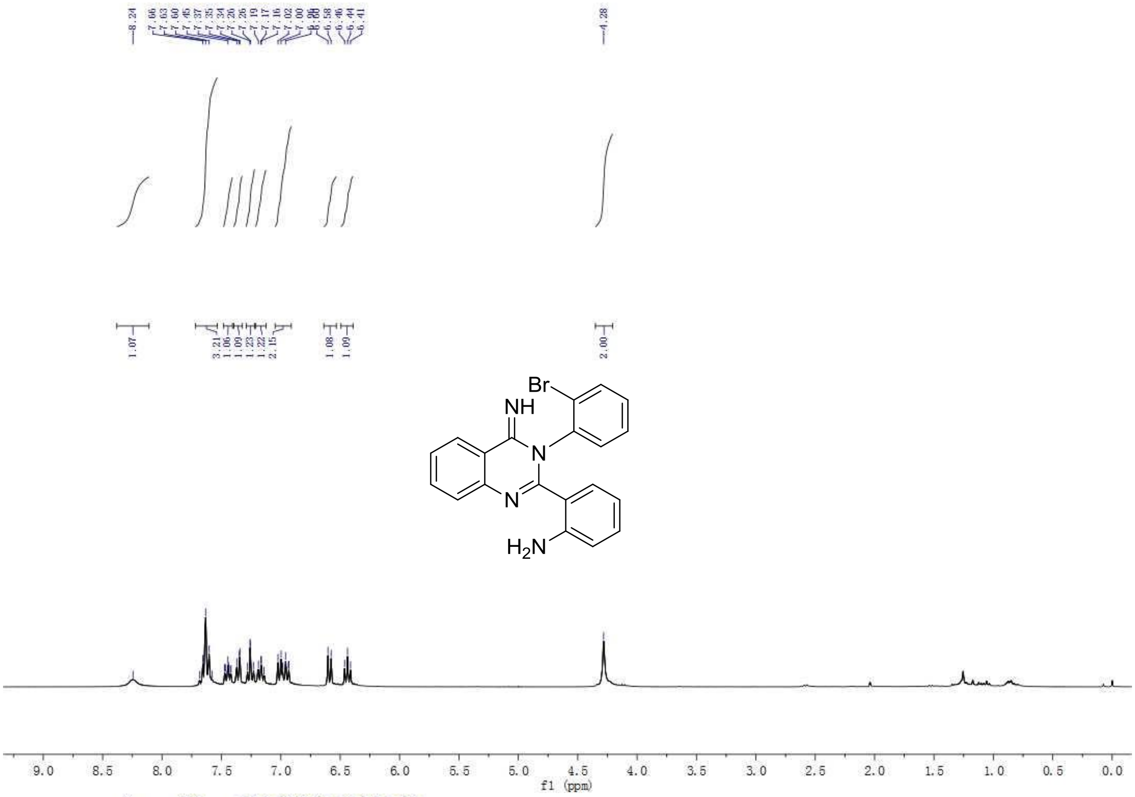

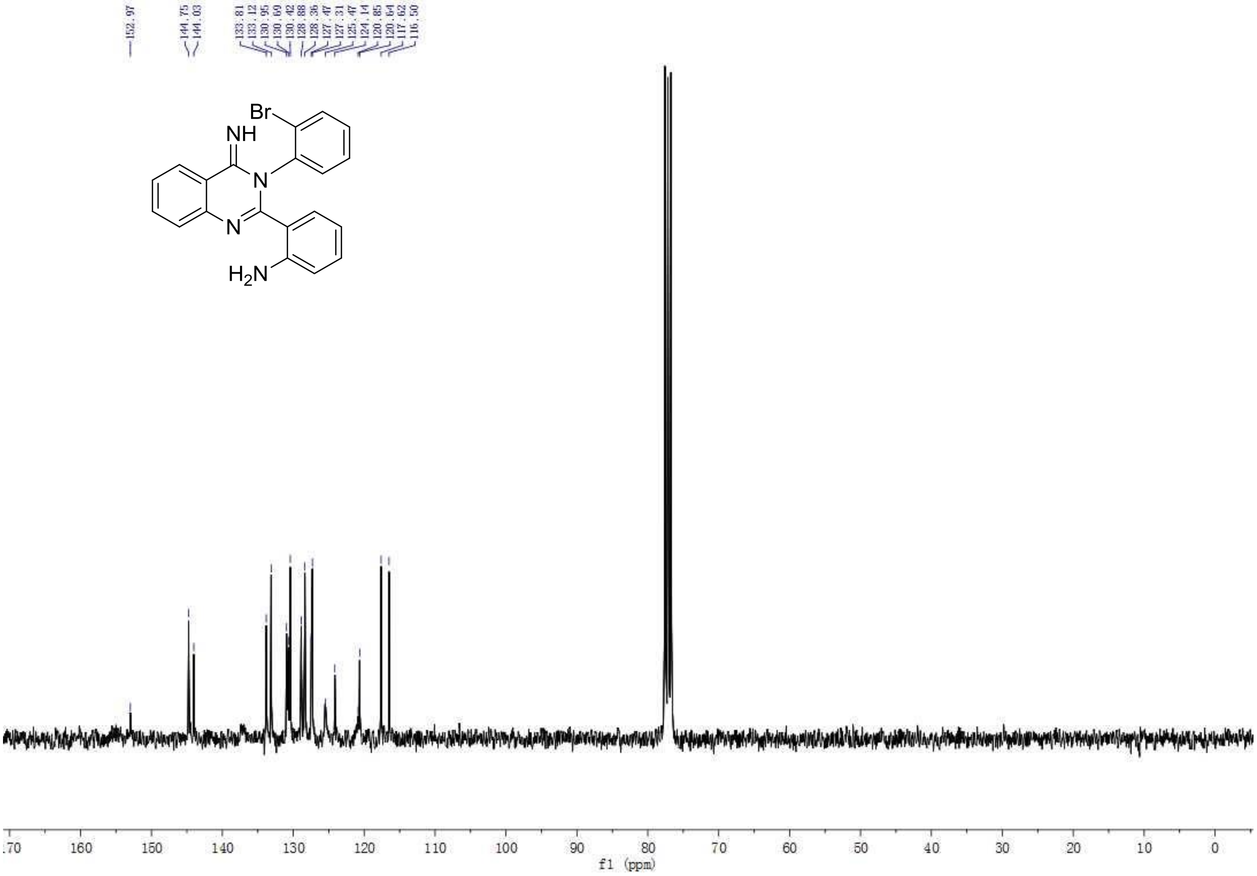

^{1}H NMR and ^{13}C NMR for compound **3a**

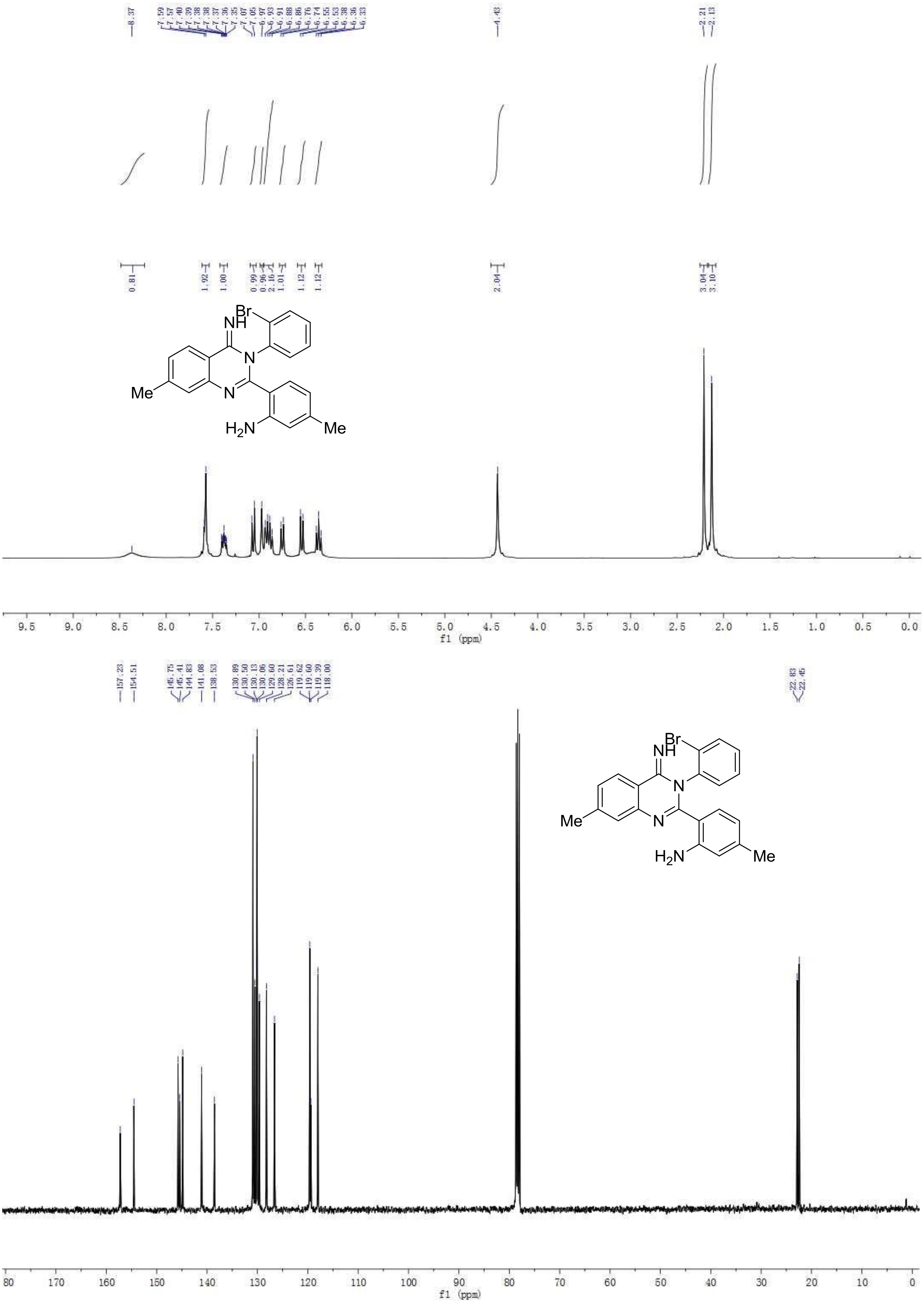

^{1}H NMR and ^{13}C NMR for compound **3b**

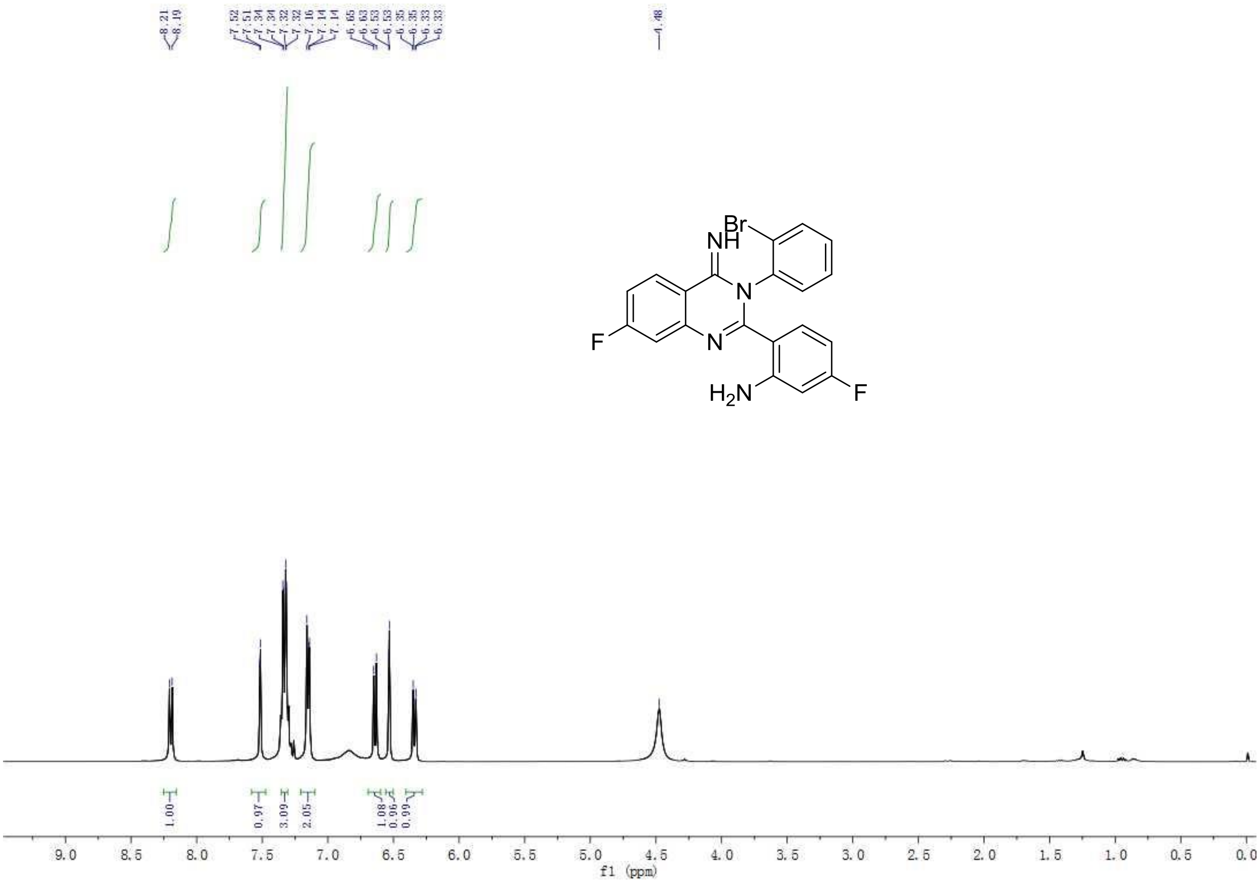

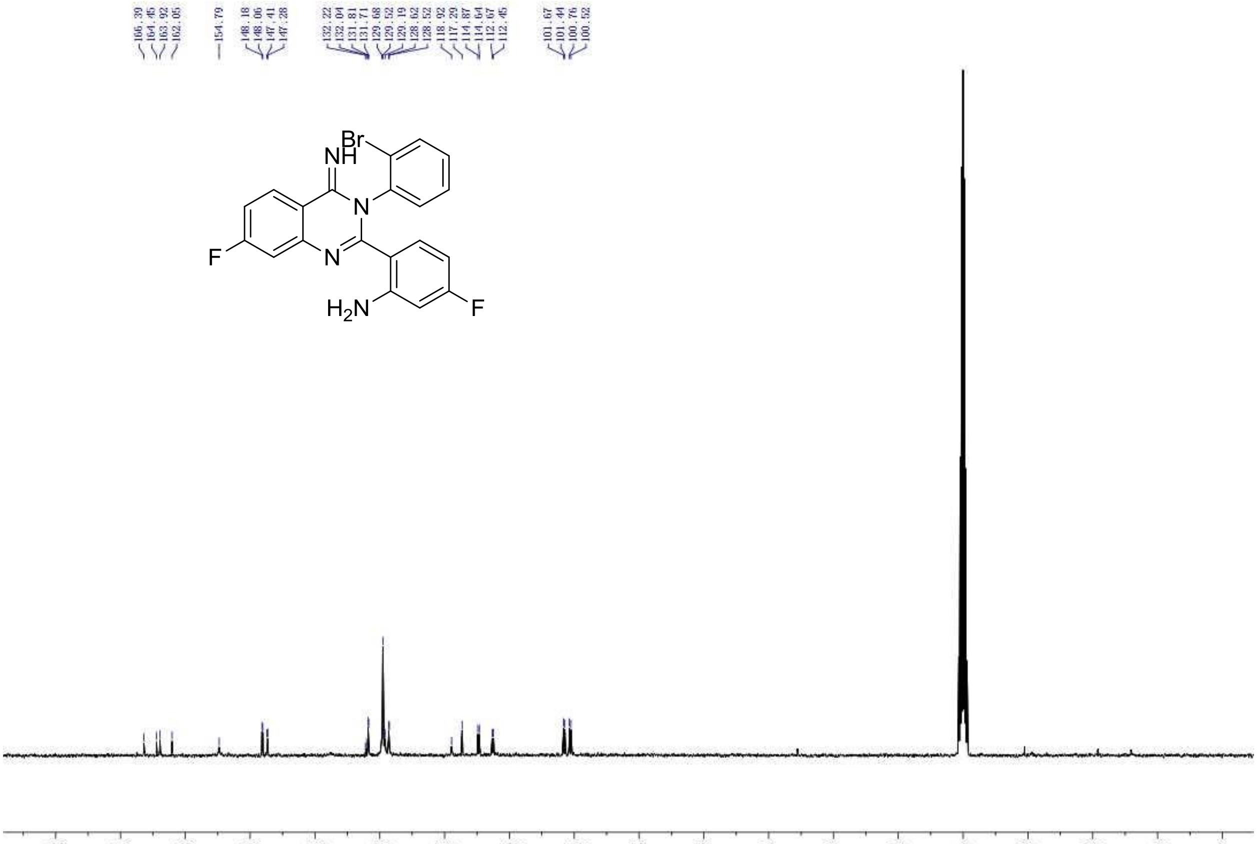

^{1}H NMR and ^{13}C NMR for compound **3c**

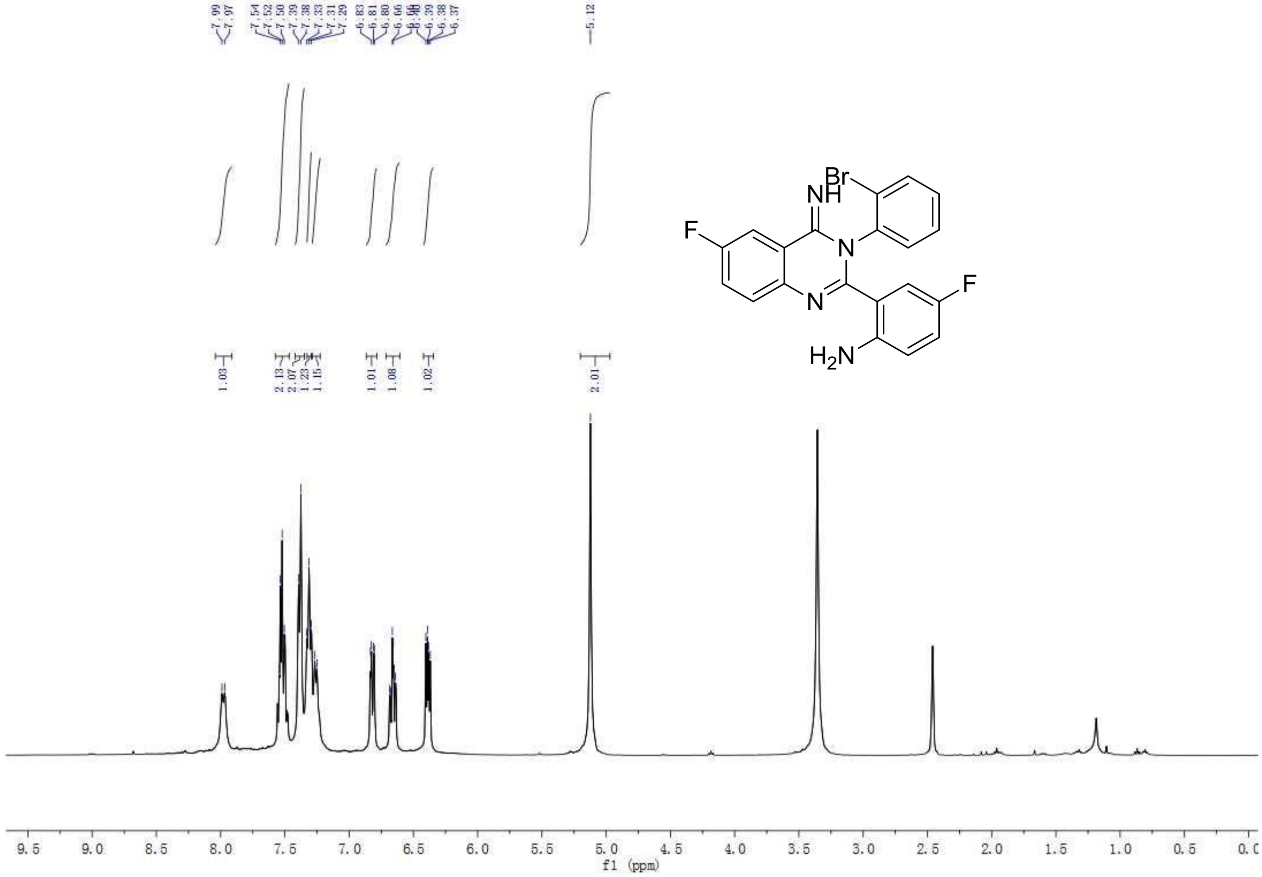

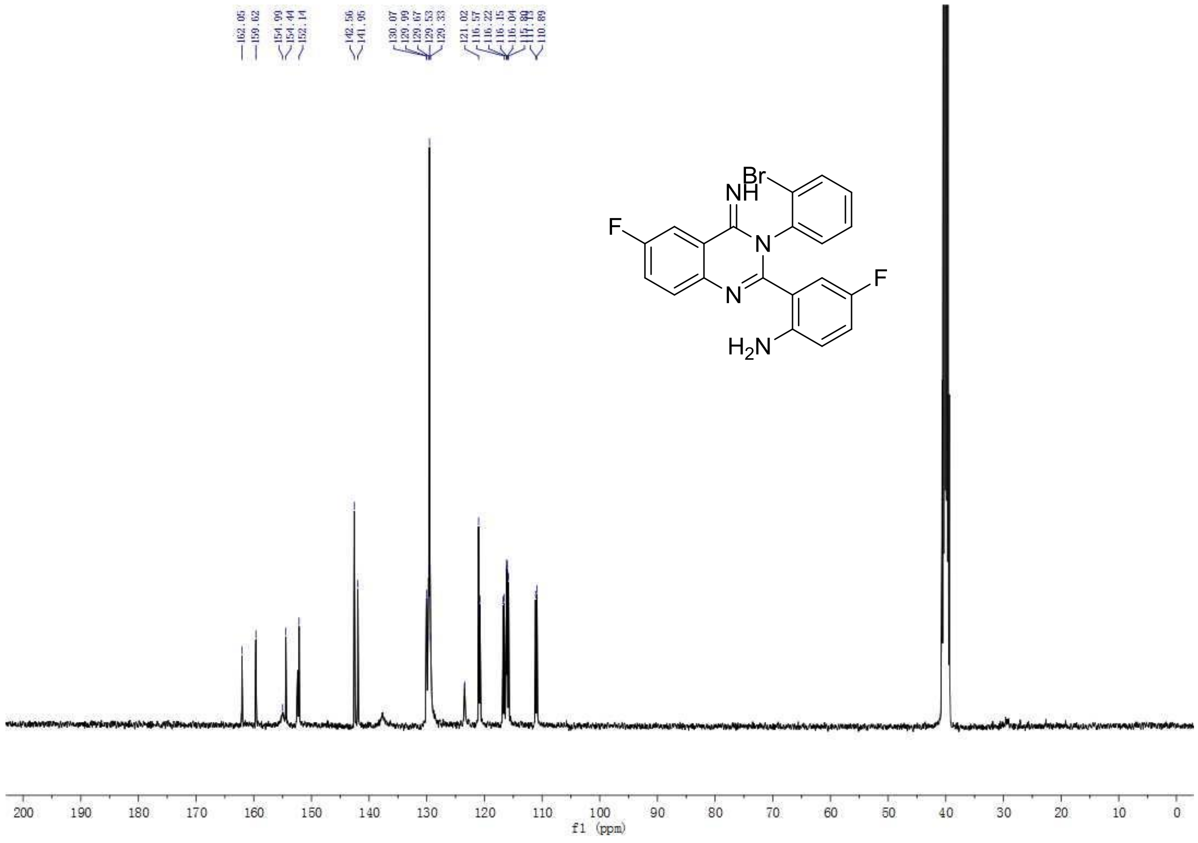

^{1}H NMR and ^{13}C NMR for compound **3d**

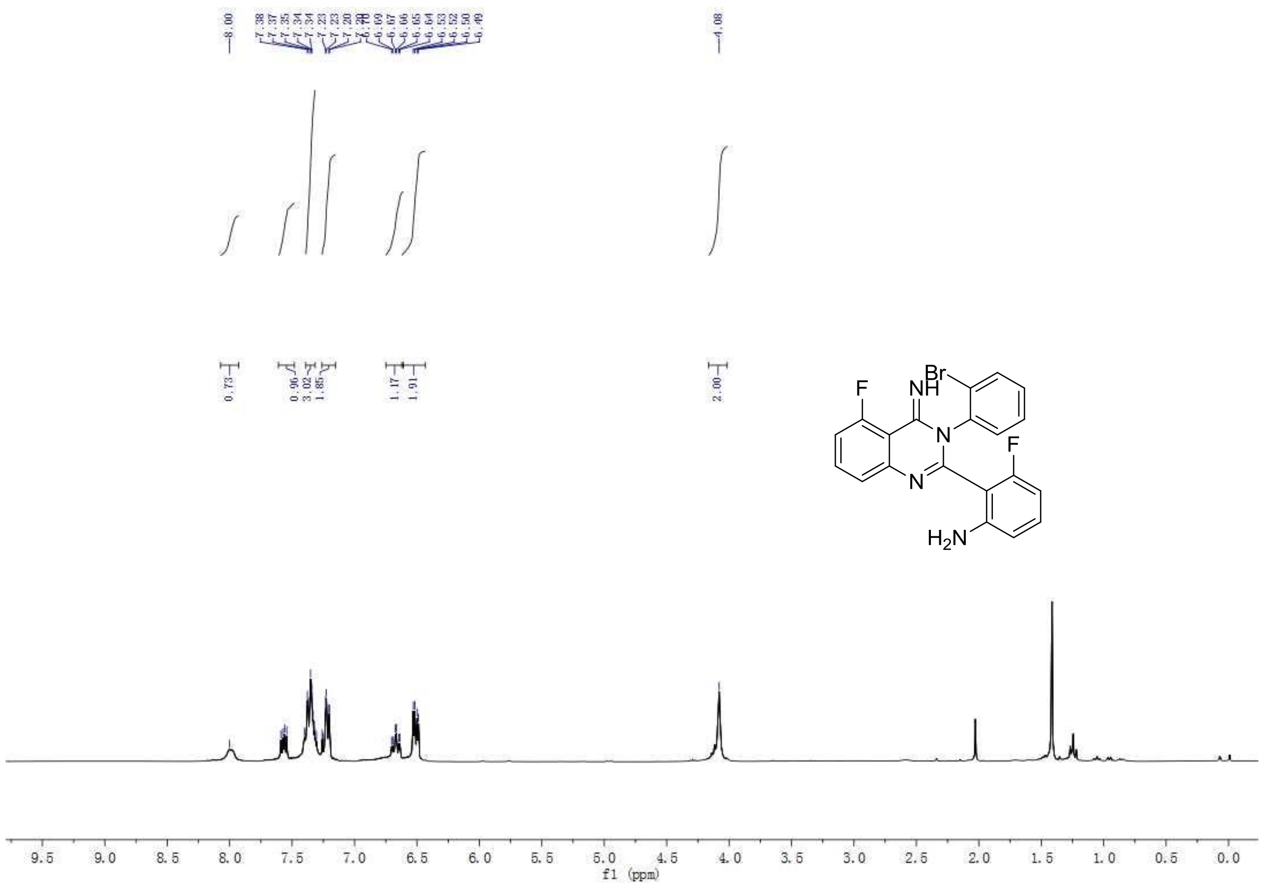

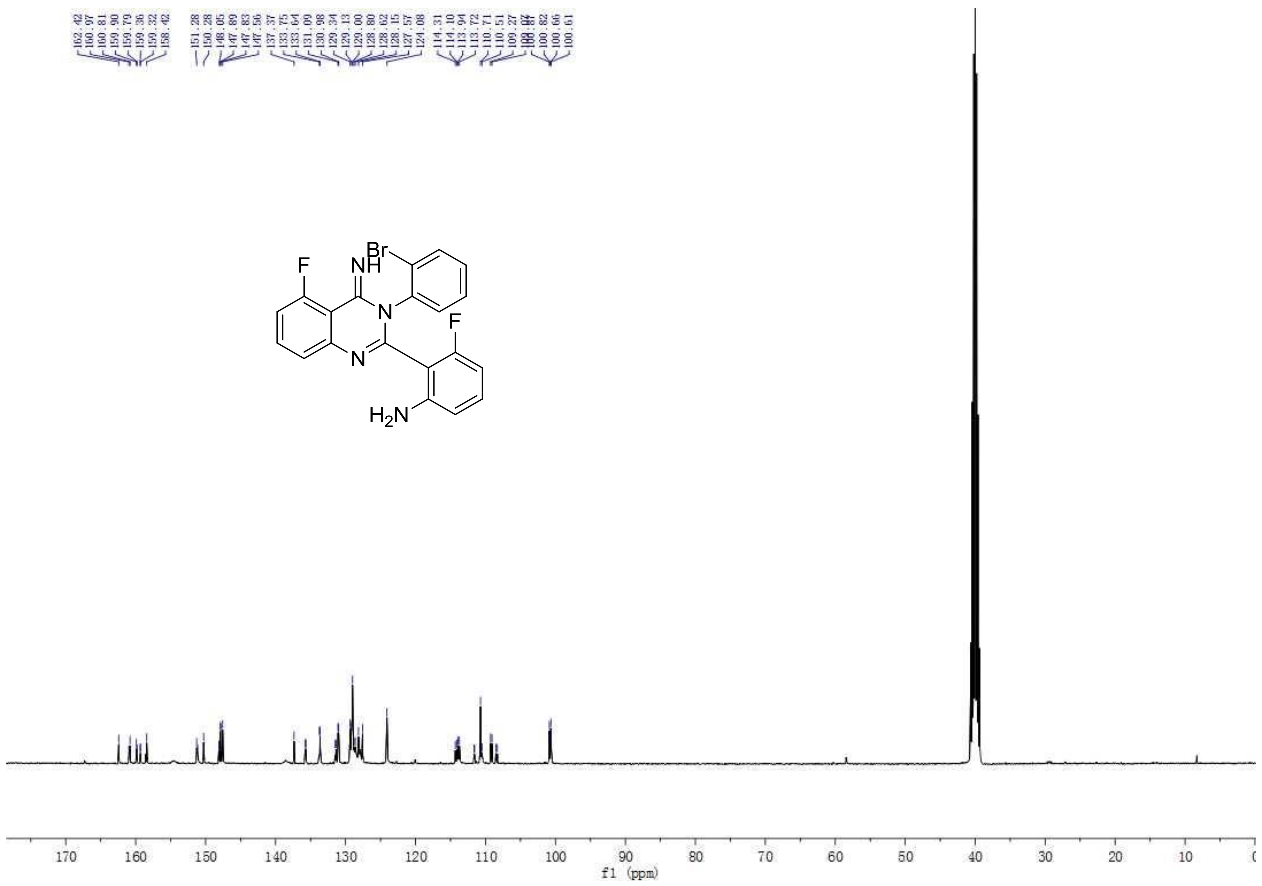

^{1}H NMR and ^{13}C NMR for compound **3e**

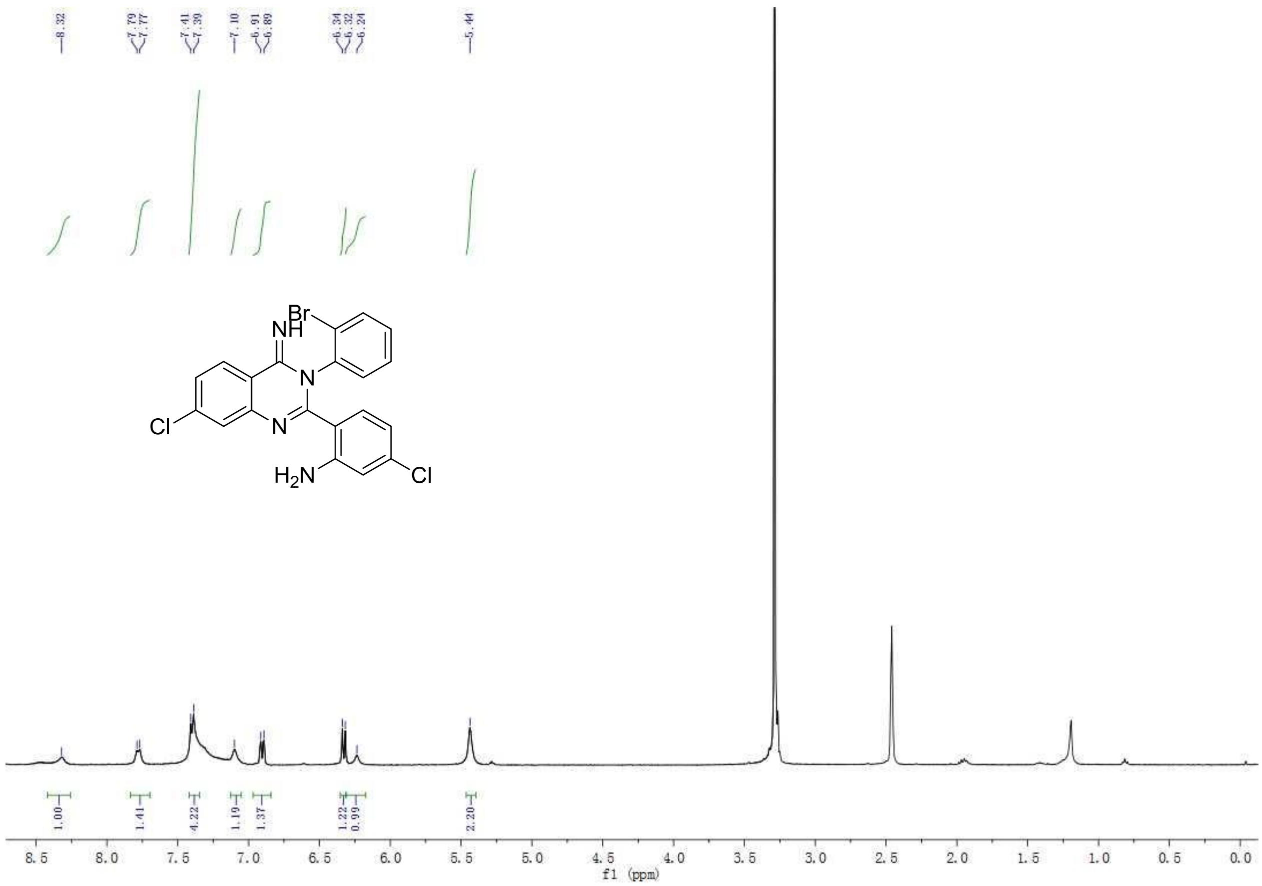

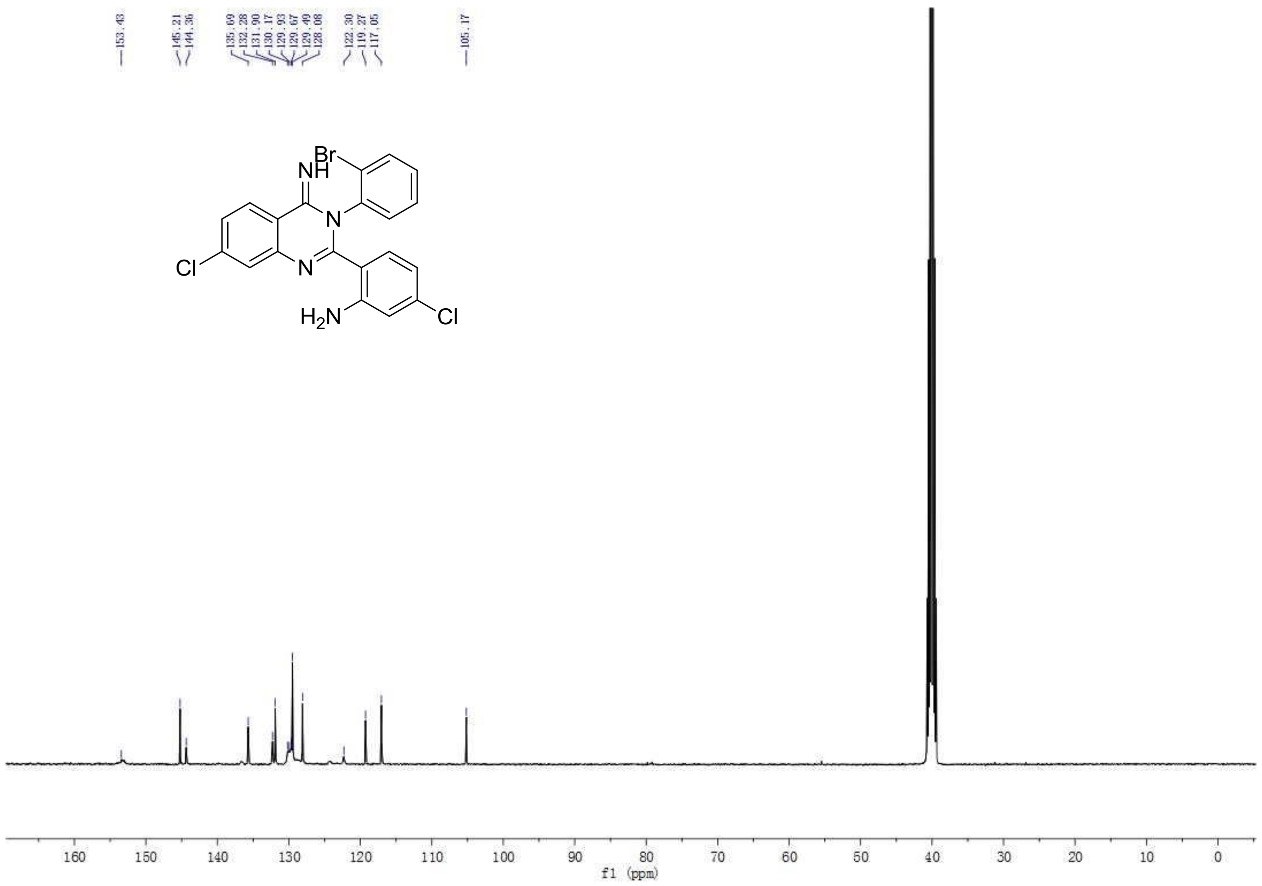

^{1}H NMR and ^{13}C NMR for compound **3f**

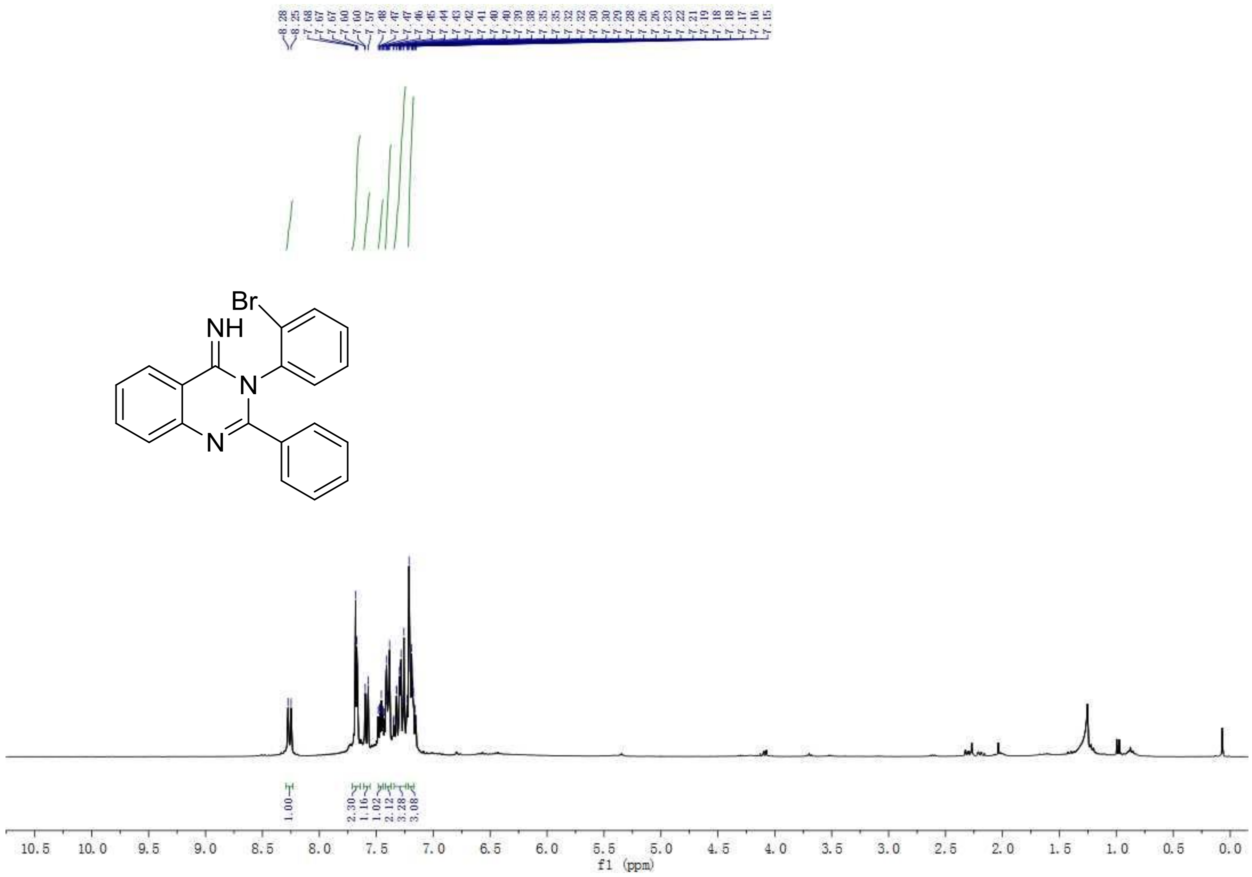

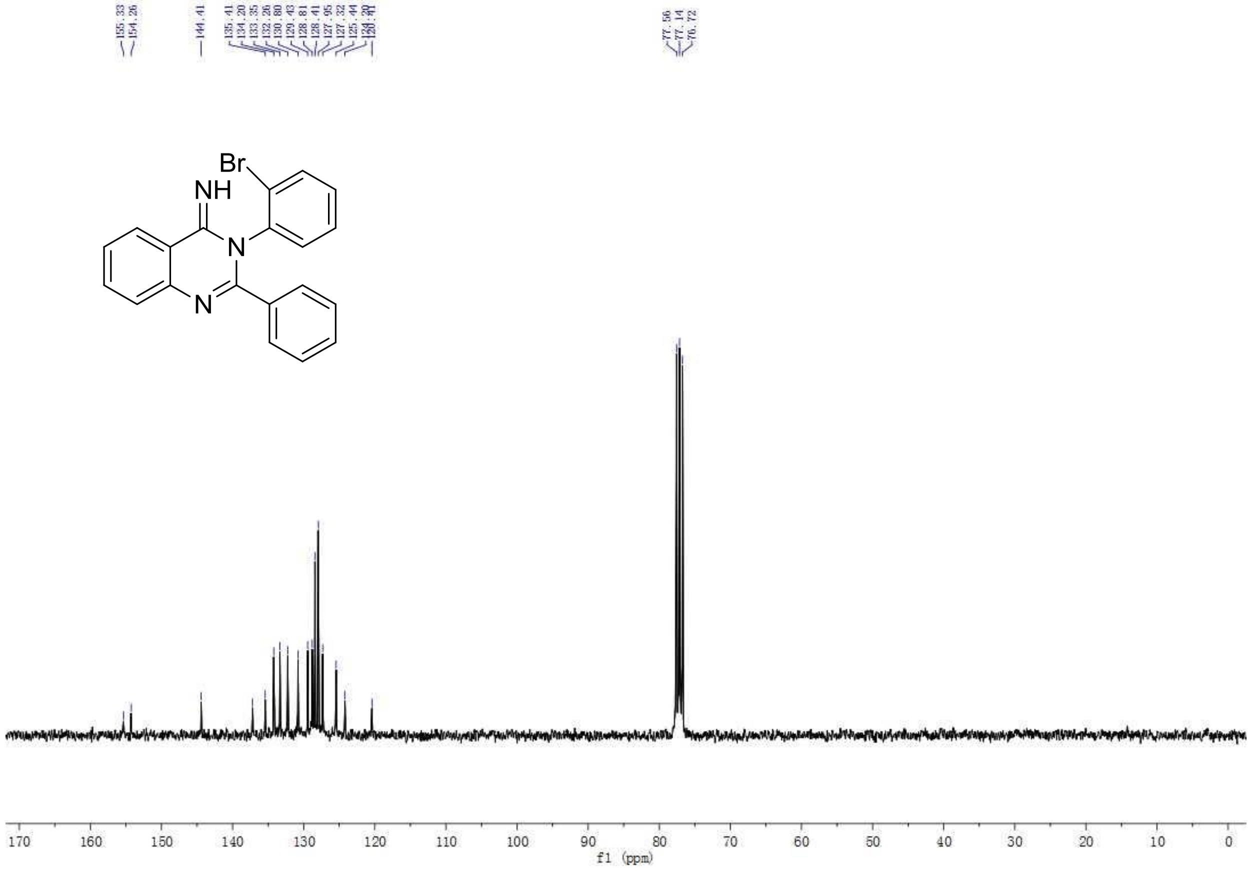

^{1}H NMR and ^{13}C NMR for compound **3g**

5). NMR spectra for compounds 4a–4g

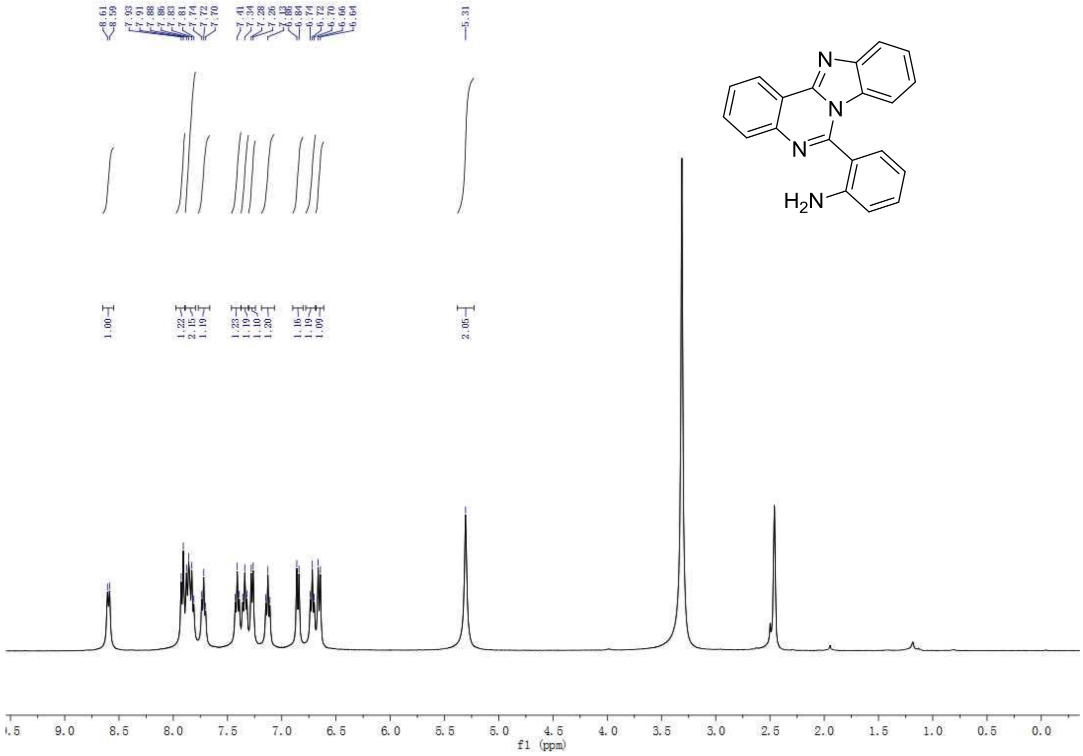

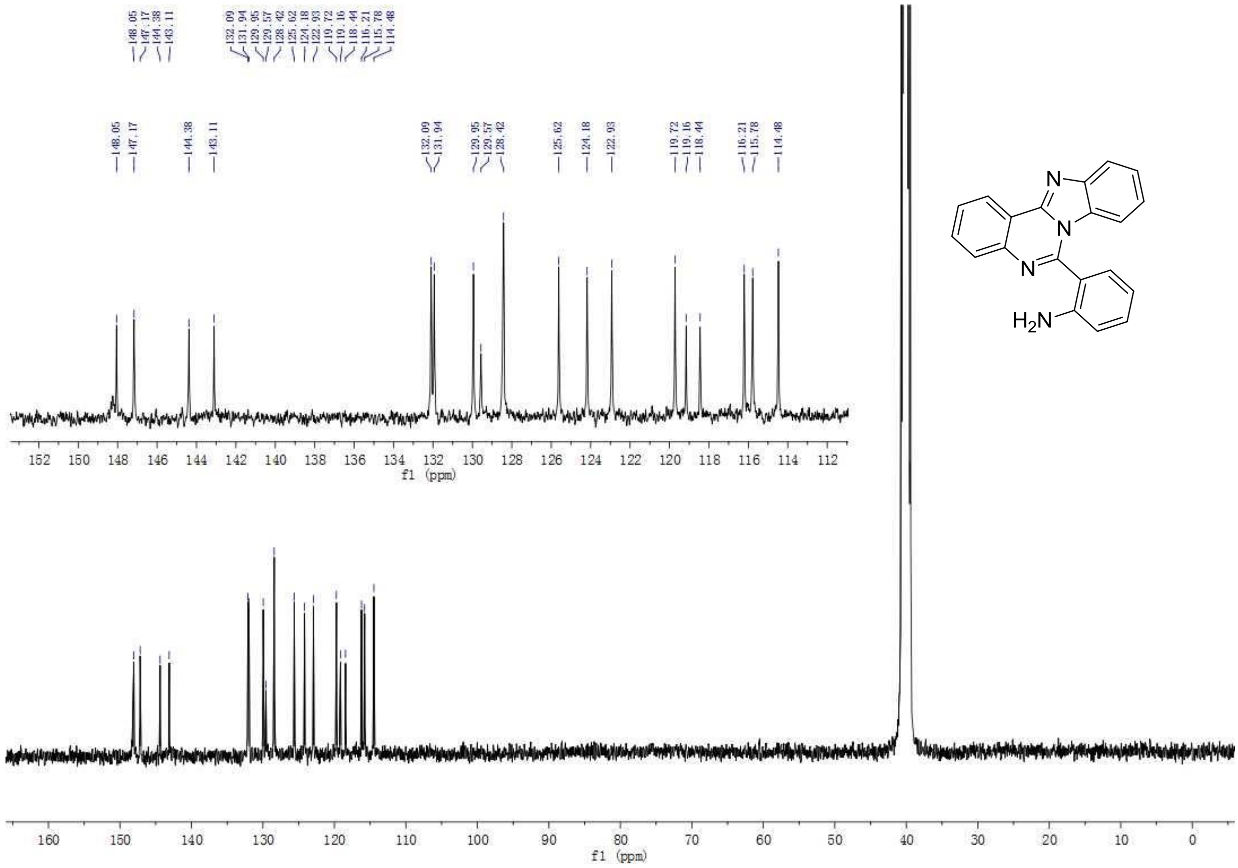

^{1}H NMR and ^{13}C NMR for compound **4a**

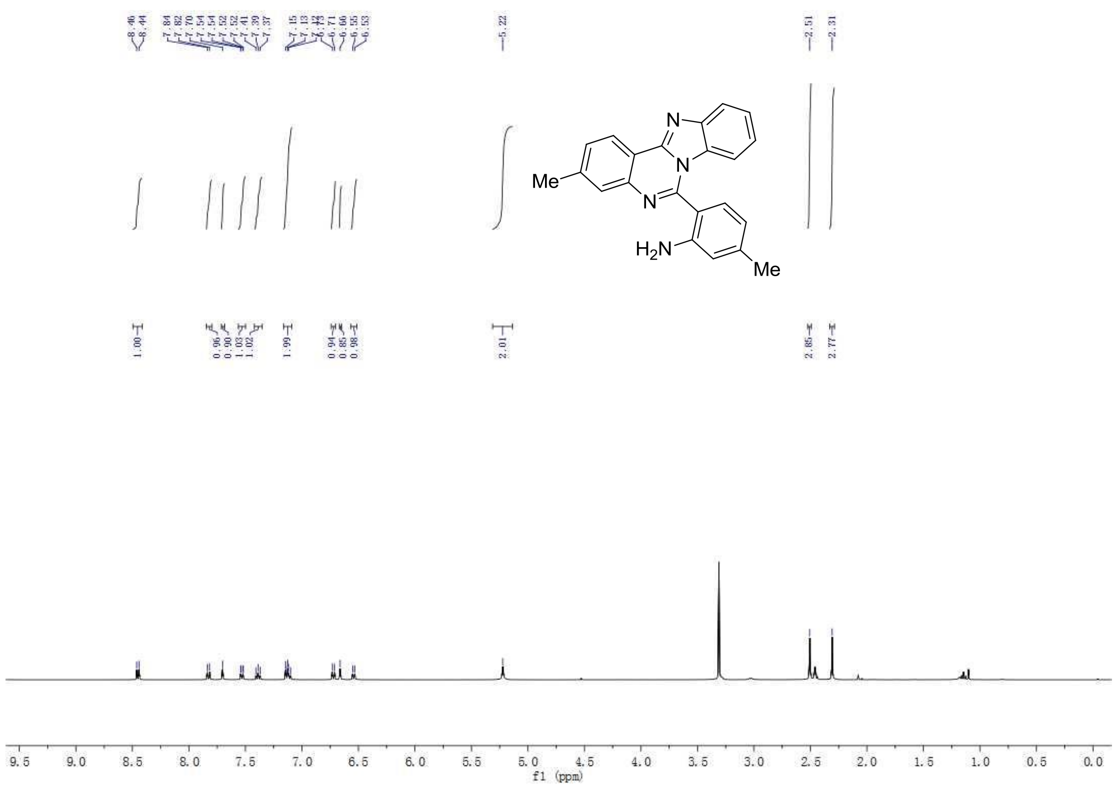

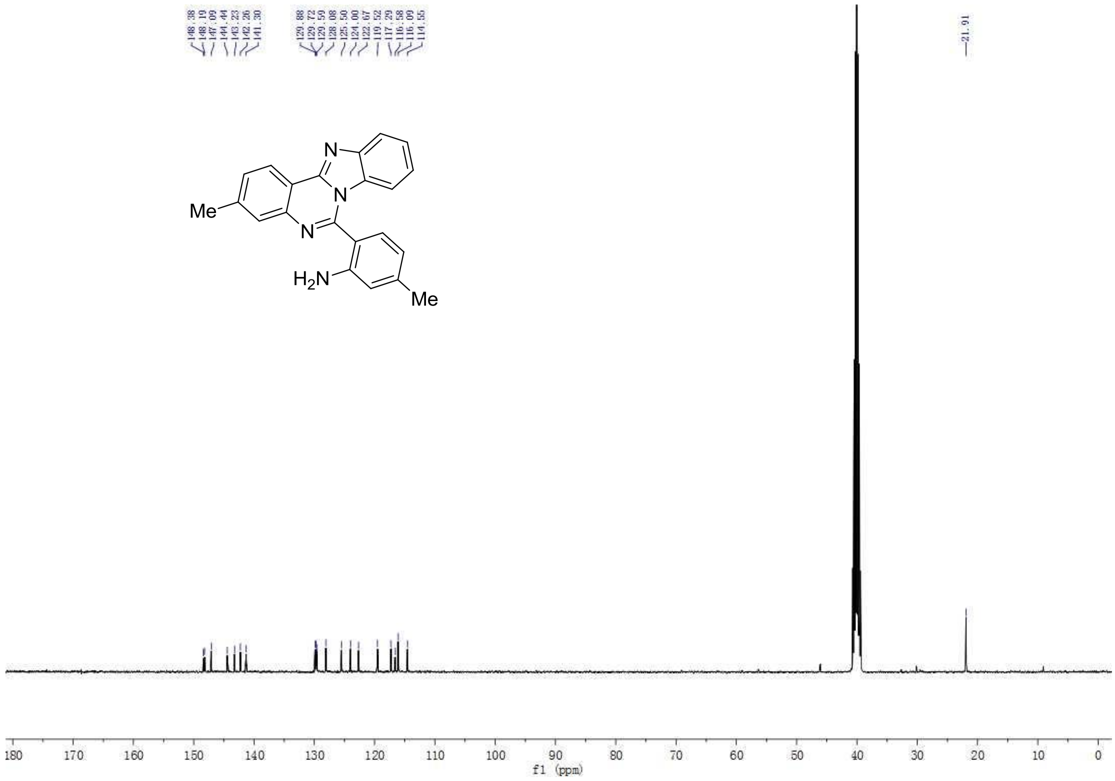

^1H NMR and ^{13}C NMR for compound **4b**

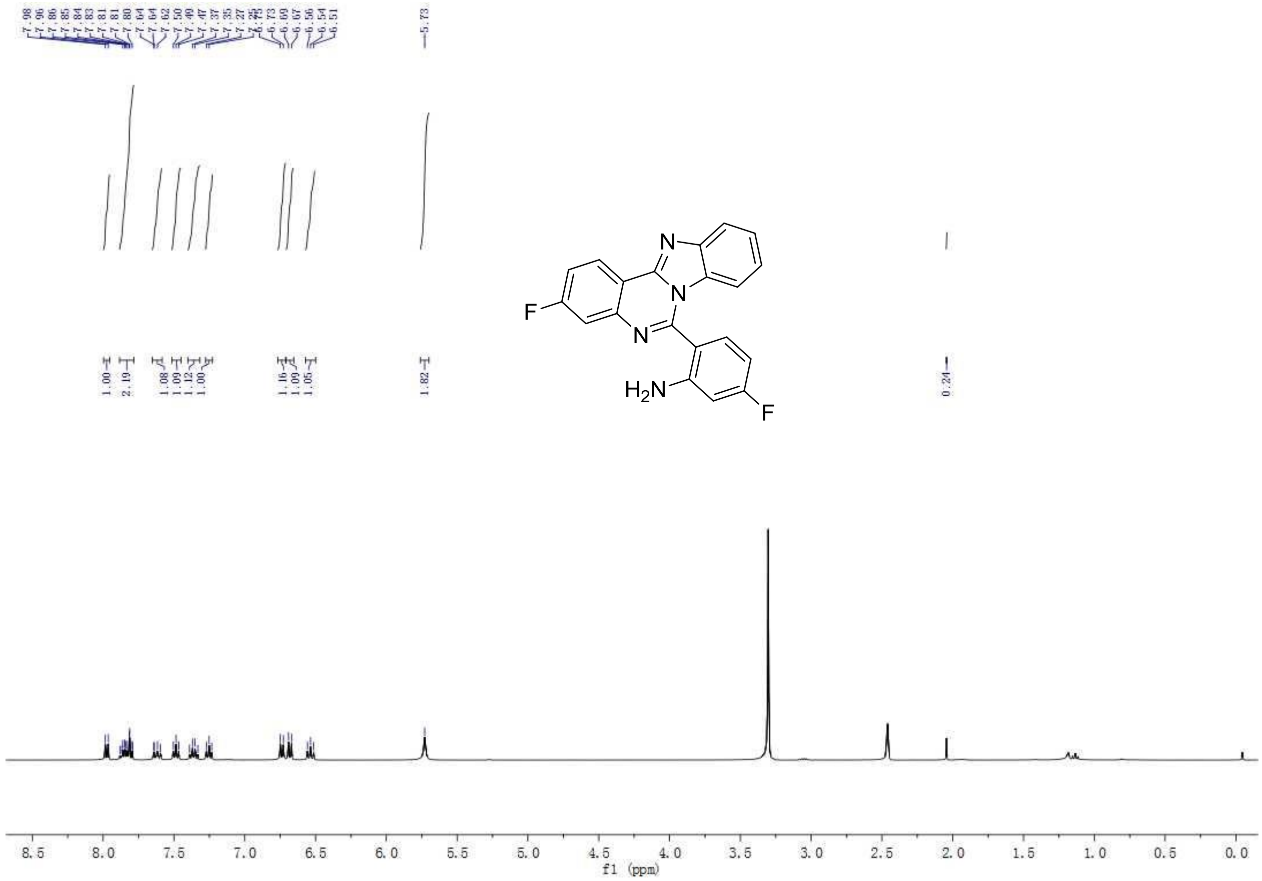

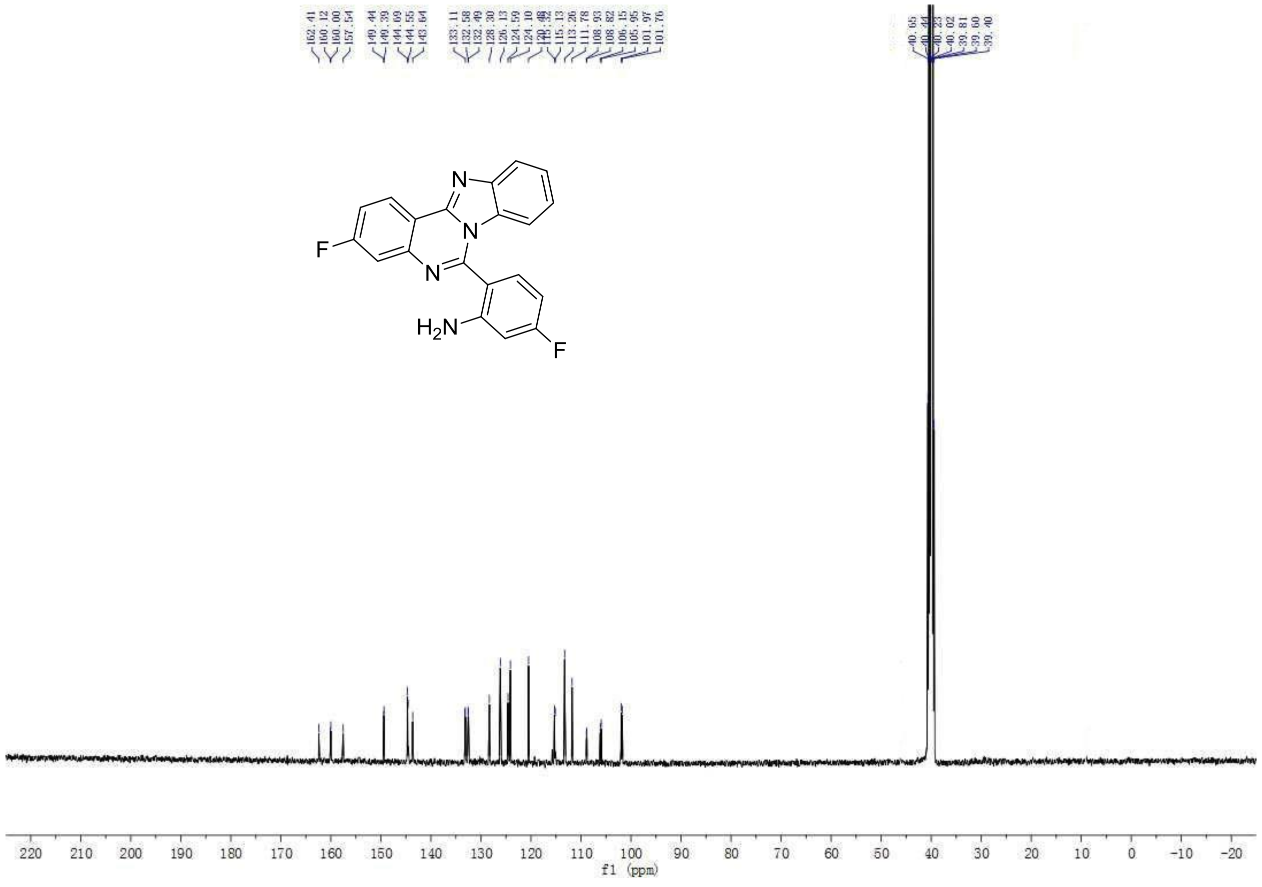

^{1}H NMR and ^{13}C NMR for compound **4c**

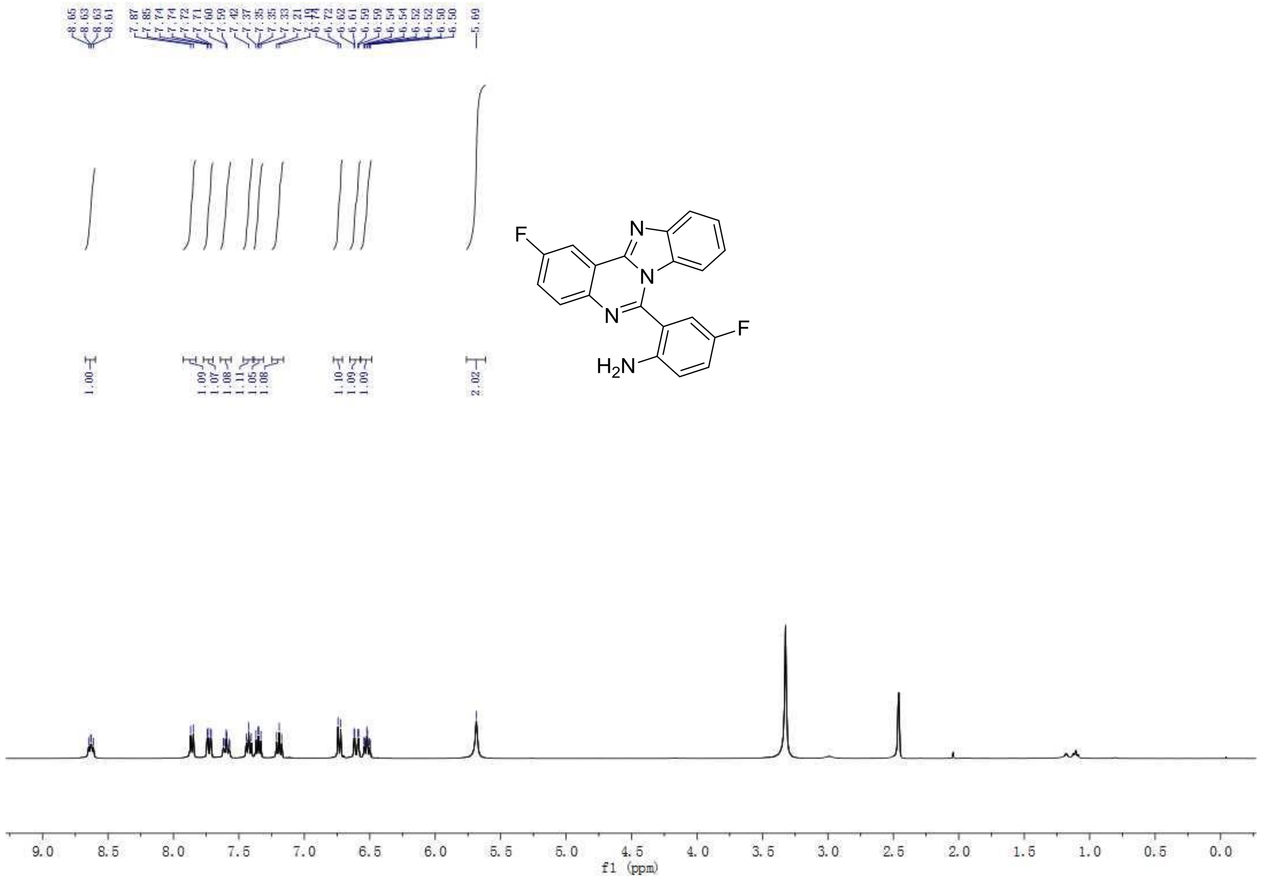

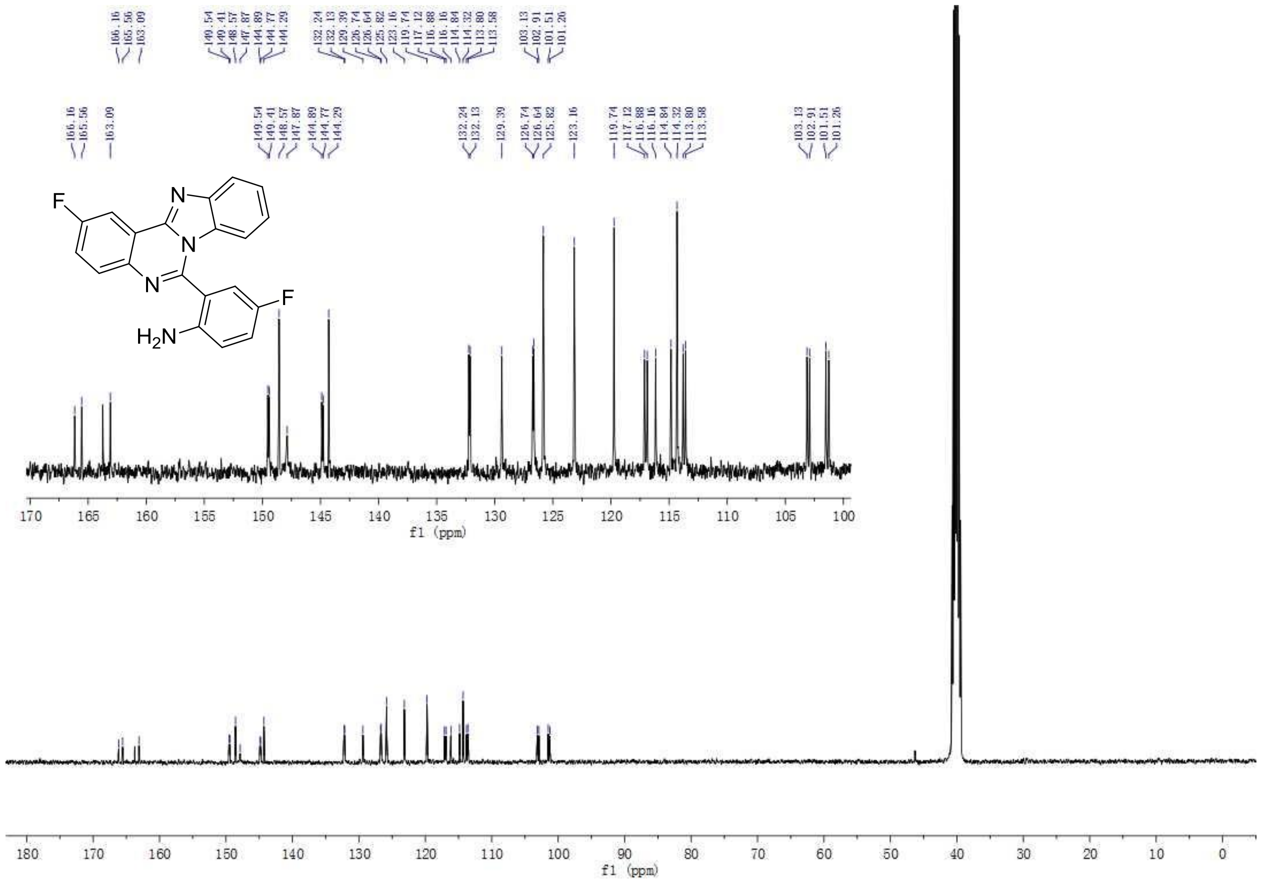

^{1}H NMR and ^{13}C NMR for compound **4d**

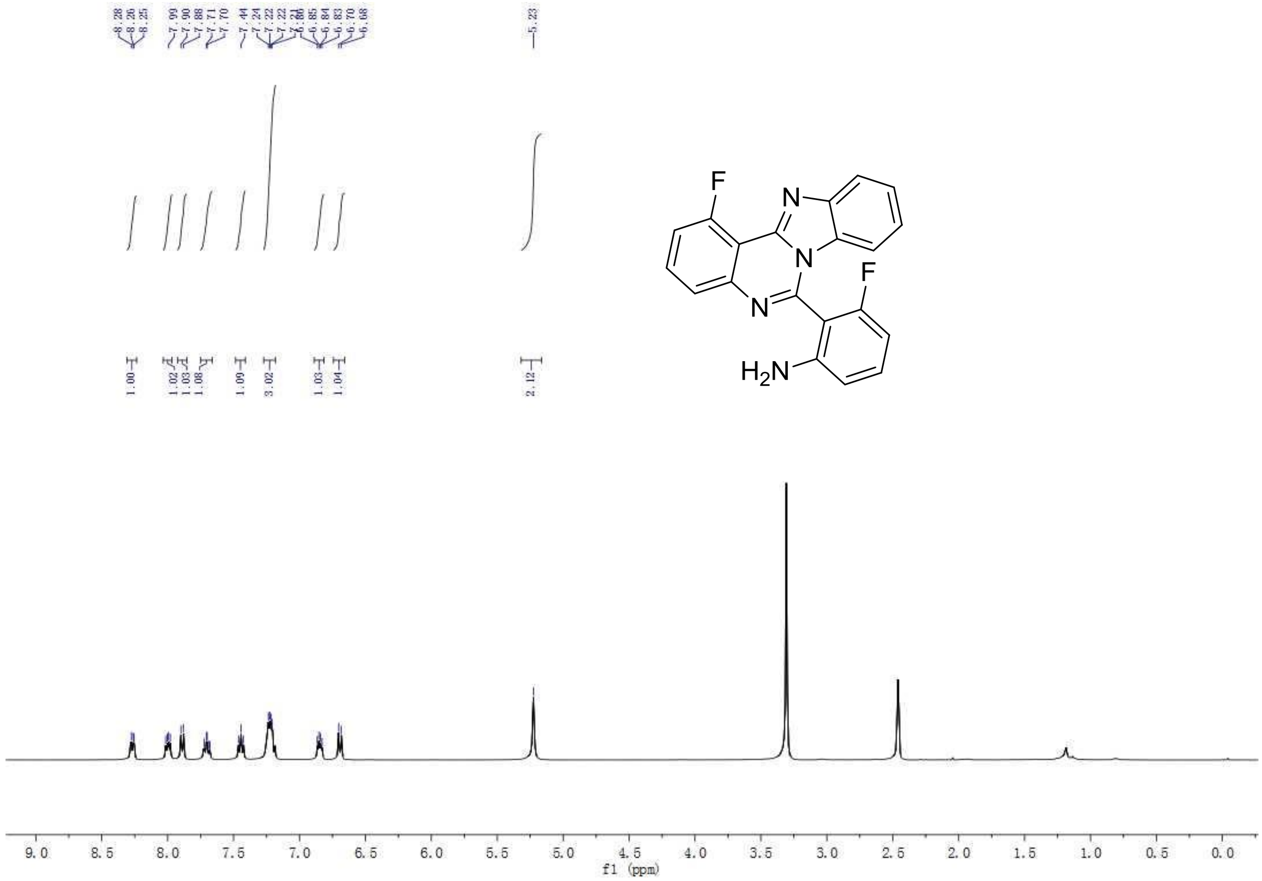

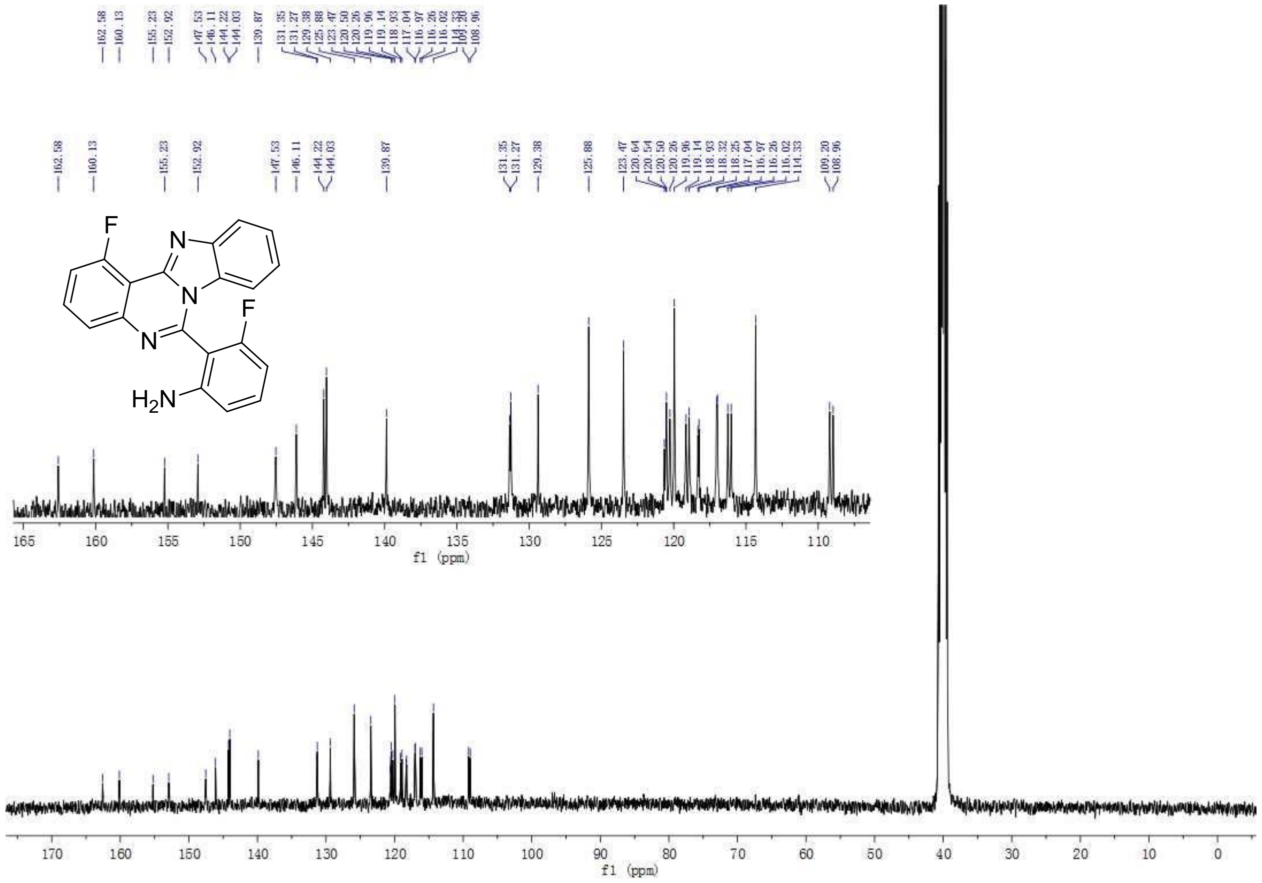

^{1}H NMR and ^{13}C NMR for compound **4e**

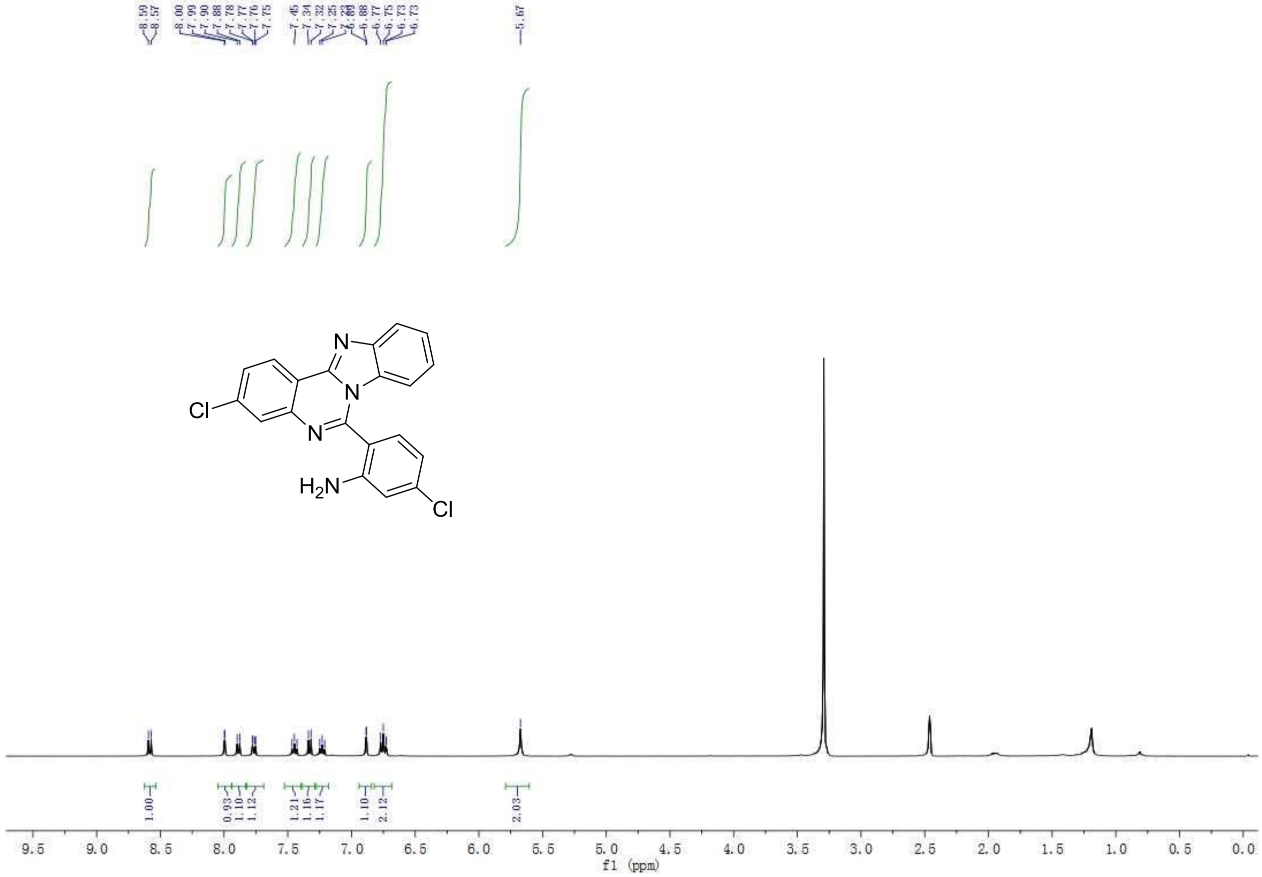

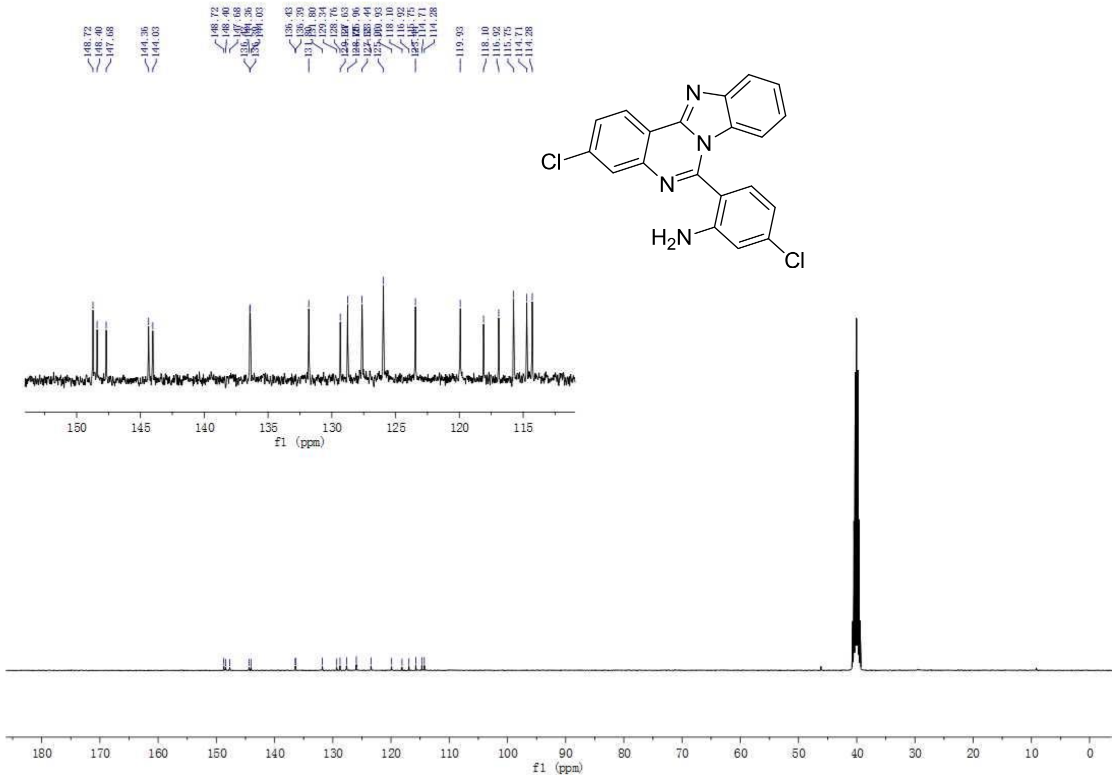

^{1}H NMR and ^{13}C NMR for compound **4f**

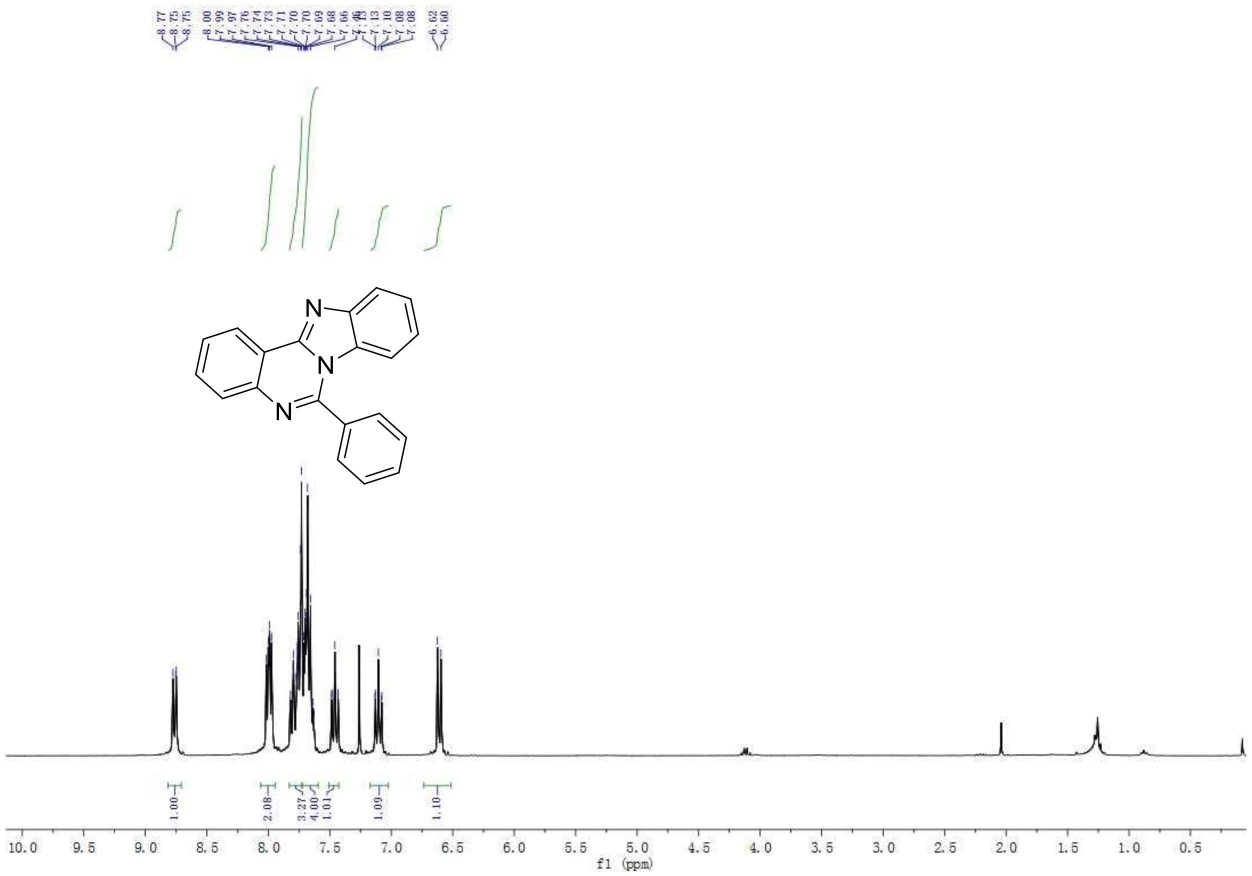

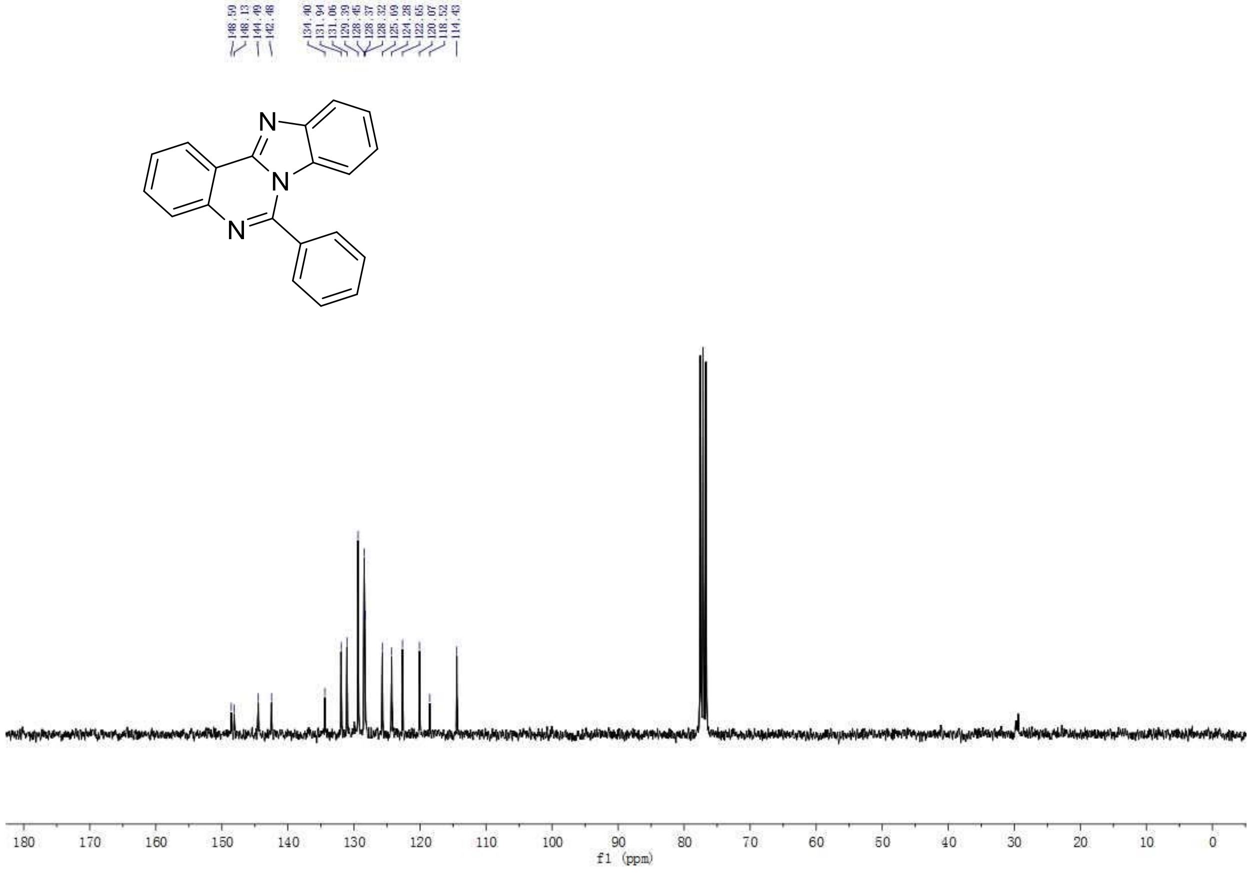

^{1}H NMR and ^{13}C NMR for compound **4g**

References:

1. Yusubov, M. S.; Maskaev, A. V.; Zhdankin, V. V. *ARKIVOC* **2011,** *i,* 370–409. doi:11-6760LR
2. Merritt, E. A.; Olofsson, B. *Angew. Chem., Int. Ed.* **2009,** *48,* 9052–9070. doi:10.1002/anie.200904689; *Angew. Chem.* **2009**, *121*, 9214-9234. doi:10.1002/ange.200904689
3. Zhdankin, V. V.; Stang, P. J. *Chem. Rev.* **2008,** *108,* 5299–5358. doi:10.1021/cr800332c
4. Zhdankin, V. V.; Stang, P. J. *Chem. Rev.* **2002,** *102,* 2523–2584. doi:10.1021/cr010003+
5. Grushin, V. V. *Chem. Soc. Rev.* **2000,** *29,* 315–324. doi:10.1039/A909041J
6. Stang, P. J.; Zhdankin, V. V. *Chem. Rev.* **1996,** *96,* 1123–1178. doi:10.1021/cr940424+
7. Krasnokutskaya, E. A.; Semenischeva, N. I.; Filimonov, V. D.; Knochel, P. *Synthesis* **2007,** *1,* 81–84. doi:10.1055/s-2006-958936
8. Yoshizawa, K.; Toyota, S.; Toda, F. *Green Chem.* **2002,** *4,* 68–70. doi:10.1039/B110437N
9. Mohamed Ahmed, M. S.; Mori, A. *Tetrahedron* **2004,** *60,* 9977–9982. doi:10.1016/j.tet.2004.08.016
10. Lou, Z.; Zhang, S.; Chen, C.; Pang, X.; Li, M.; Wen, L. *Adv. Synth. Catal.* **2014,** *356,* 153–159. doi:10.1002/adsc.201300728
